# A study on the effects of a whole grain diet combined with short duration exercise on postprandial glucose levels in overweight and obese adults

**DOI:** 10.3389/fnut.2026.1886129

**Published:** 2026-07-01

**Authors:** Yinfeng Wang, Leqin Chen, Lijuan Yao, Ruiwu Guo, Yuting Chen, Ruirui Shi

**Affiliations:** School of Physical Education, Shanxi Normal University, Taiyuan, Shanxi, China

**Keywords:** continuous glucose monitoring, grain powder, moderate-to-vigorous exercise, overweight and obesity, postprandial glycemia

## Abstract

**Objective:**

Postprandial glycemic excursions represent a critical determinant of metabolic health in individuals with overweight or obesity. Previous studies have demonstrated that moderate-to-vigorous exercise lasting at least 5 min, initiated 30 min after breakfast, significantly attenuates postprandial glycemic responses and reduces peak glucose levels. However, whether this effect remains consistent across various grain powder based meals is still unclear. This study therefore examined the exercise diet interaction to determine whether 5 min of moderate-to-vigorous exercise performed 30 min after breakfast could elicit a distinct glucose lowering effect depending on the dietary composition.

**Methods:**

This study adopted a within subject, fixed sequence, repeated measures intervention design. A total of 43 adults with overweight or obesity were enrolled and completed a 15 day dietary and exercise intervention trial. Throughout the experimental period, participants consumed a test breakfast daily consisting of a designated grain powder served with one boiled egg; meanwhile, their remaining dietary intake was not controlled, and they were instructed to maintain their habitual lifestyles. The five grain powders—tartary buckwheat flour, millet flour, oat flour, yam flour and soy milk powder were commercially available products (Joyoung brand) procured by the research team. On the third day of each 3 day breakfast intervention phase, participants performed 5 min of moderate-to-vigorous exercise 30 min after breakfast consumption. The exercise routine comprised jumping jacks, lateral squats, shadow punches, under leg claps, and lateral shuffles. Each movement was executed for four 8 count phrases synchronized with Tabata style music. Demonstration videos of the exercise routine were recorded by the research staff, who also provided instruction and supervised the exercise sessions. Statistical analyses, including non-parametric tests and generalized estimating equations generalized estimating equations (GEE), were performed using SPSS software, and data visualization was conducted using GraphPad Prism.

**Results:**

This study analyzed the main effects of grain powder type and exercise, as well as their interaction, on 2-h postprandial glycemic (2-h PBG) responses using a GEE model. Model effect tests indicated that the type of grain powder significantly influenced postprandial glycemic parameters, including the incremental area under the curve (iAUC; Wald χ^2^ = 64.997, *p* < 0.001), peak glucose increment (Wald χ^2^ = 45.989, *p* < 0.001), and glycemic variability (GV) (Wald χ^2^ = 86.352, *p* < 0.001). Neither the main effect of exercise nor the powder–exercise interaction was statistically significant (all *p* > 0.05). Soy milk powder elicited the lowest iAUC (51.494 ± 6.335, 95% CI: 39.078–63.911), which was significantly lower than those following the consumption of oat powder (107.421 ± 10.591, 95% CI: 86.662–128.179), millet powder (95.319 ± 10.078, 95% CI: 75.567–115.071), and tartary buckwheat powder (93.542 ± 8.031, 95% CI: 77.801–109.283; all *p* < 0.01). Additionally, soy milk powder resulted in the lowest peak glucose increment (1.194 ± 0.101, 95% CI: 0.997–1.391) and GV (0.068 ± 0.004, 95% CI: 0.060–0.076). By contrast, yam powder yielded a significantly lower overall glucose area under the curve (AUC; 746.773 ± 13.669, 95% CI: 719.982–773.563) compared with tartary buckwheat powder, millet powder, and oat powders (all *p* < 0.05).

**Conclusion:**

Within the present study context, the type of grain powder was identified as the primary factor influencing postprandial glycemia in adults with overweight or obesity, whereas neither the short bout of moderate-to-vigorous exercise nor the exercise–powder interaction achieved statistical significance. Soy milk powder elicited the lowest iAUC, peak glucose increment, and GV, a favorable glycemic profile largely attributable to its low carbohydrate and high protein composition. These findings suggest that incorporating high protein, low glycemic index options such as soy milk powder into breakfast may favorably modulate acute postprandial glycemic responses.

## 1 Introduction

In 2022, the World Health Organization reported that 2.5 billion adults (aged ≥18 years) worldwide lived with overweight, of whom 890 million had obesity (1). In recent years, the prevalence of overweight and obesity has continued to rise in China, with national data indicating that 34.3% of adults have overweight and 16.4% have obesity (2). Obesity is recognized both as an independent chronic disease and as a precipitating factor for multiple other chronic conditions (3); in particular, it is a well established risk factor for hyperglycemia and diabetes (4). It has become a major public health concern in China, ranking as the sixth leading risk factor for mortality and disability (5). Genetic predisposition, dietary patterns, physical activity, psychological status, sleep behaviors, comorbidities, medication use, and socio-environmental factors are key determinants of obesity development (6). Among these, the interaction between diet and physical activity warrants particular attention.

In contemporary society, substantial societal pressures and fast paced lifestyles have led to irregular dietary habits among adults and adolescents, who frequently skip breakfast even when food is available, primarily due to time constraints (7). As the first meal of the day, breakfast composition directly influences the initial characteristics of postprandial glycemic excursions. Elevated postprandial glycemia is recognized as a risk factor for metabolic diseases, including cardiovascular disease, obesity, and metabolic syndrome; thus, maintaining postprandial glucose levels within or near the normal range reduces the risk of metabolic disease progression (8, 9). Previous studies have demonstrated that postprandial exercise, specific exercise modalities, and the timing of activity can improve postprandial glycemic responses (10–14). Short duration exercise, for instance, has been shown to activate the sympathetic nervous system, improve vascular function, reduce postprandial and peak glucose levels (15), and preserve glucose homeostasis. These exercise interventions—including walking, stair climbing, and resistance exercise—have been validated under both laboratory and free living conditions (16, 17). Furthermore, a prospective cohort study demonstrated that engaging in more than 5 min of moderate-to-vigorous physical activity daily significantly reduces the risk of diabetes (18). However, current research has predominantly focused on glycemic characteristics in patients with diabetes, with insufficient attention paid to adults with overweight or obesity who have not yet received a diabetes diagnosis. Moreover, existing studies on glycemic profiles have largely examined the independent effects of diet or exercise, with a lack of systematic analysis regarding their interactive or synergistic effects.

Continuous glucose monitoring (CGM) can guide dietary choices, promote healthy lifestyles (19, 20), and facilitate reductions in mean and fasting glucose concentrations (21). By utilizing smartphone connected applications, CGM continuously records glucose levels at 5-min intervals (22), facilitating the characterization of fasting and postprandial glycemic responses (23) and the detection of abrupt glycemic changes following dietary or physical activity interventions (24). By capturing dynamic glucose curves, CGM reflects the influence of various factors on glycemic fluctuations, provides insights into the glycemic response patterns underlying glucose homeostasis, and enables a more comprehensive evaluation of the modulatory effects of grain powders and exercise on overall glycemic control. Furthermore, prior research has confirmed that CGM derived measurements obtained during exercise demonstrate high concordance with reference glucose values in young adults with overweight or obesity (25). Therefore, this study employed CGM technology to investigate the postprandial glycemic responses to five different grain powders and to characterize the corresponding glycemic profiles following an exercise intervention.

## 2 Aim and hypotheses

The aim of this study was to examine the acute effects of grain powder consumption combined with exercise on postprandial glycemic responses. We hypothesized that (i) different types of grain powder would elicit distinct postprandial glycemic responses; (ii) a short-duration bout of moderate- to-vigorous exercise would reduce postprandial glucose levels; and (iii) the glycemic effects of this postprandial exercise would vary depending on the specific type of grain powder consumed at breakfast.

## 3 Methods

### 3.1 Participants

This study was a 15-day behavioral and dietary intervention experiment designed to investigate the effects of diet combined with exercise on glucose-lowering outcomes in adults with overweight or obesity. Participants were recruited via online advertisements from December 1, 2025, to December 20, 2025. Adults with a body mass index (BMI) ≥ 24 kg/m^2^ were eligible for inclusion. Individuals with underlying metabolic or skin diseases, those taking medications affecting sleep quality, smokers, and those with regular exercise habits were excluded. All eligible participants underwent in-person screening, were fully informed of the study protocol, and provided written informed consent prior to the start of the experiment. The study protocol was approved by the Ethics Committee of XX Normal University (Approval No.: 2024–1,002).

### 3.2 Study overview

During the initial session, baseline data, including age, height, weight, body composition, and medication use, were collected from all participants via a questionnaire. Prior to the experiment, participants were required to maintain an overnight fast after their evening meal. On the morning of the experimental day, they underwent a physical examination and were fitted with a CGM device. Standardized multigrain powders were then distributed to the participants, who subsequently received training on the preparation methods and exercise protocols. Following these procedures, participants consumed a test breakfast consisting of the designated multigrain powder and one boiled egg. For lunch and dinner, they were instructed to maintain their habitual dietary patterns. Participants were required to upload their lunch and dinner dietary records via an online platform and to finish their evening meal before 8:00 p.m. This design aimed to simulate authentic daily free-living conditions, minimize the burden of strict dietary control, prevent extreme variations in daily caloric intake from confounding the subsequent day’s basal metabolic state, and maximize participant compliance.

During the intervention period, participants cycled through the five different types of multigrain powder, changing the powder type every 3 days. They were required to consume the test breakfast within a 15-min window. The test sequence was as follows: tartary buckwheat flour, millet flour, oat flour, yam flour, and soy milk powder. On the third day of each 3-day intervention phase, participants performed 5 min of moderate-to-vigorous exercise 30 min after breakfast, ensuring the activity was completed prior to the anticipated postprandial glucose peak. Previous studies have indicated that postprandial glucose peaks typically occur 30 to 60 min after meal ingestion (26), and that exercising before the glucose peak confers greater metabolic benefits than exercising later (27). If participants experienced hunger 2 h postprandial, they were permitted to consume an additional snack for energy supplementation. Upon study completion, participants received appropriate financial compensation.

**Figure 1:**
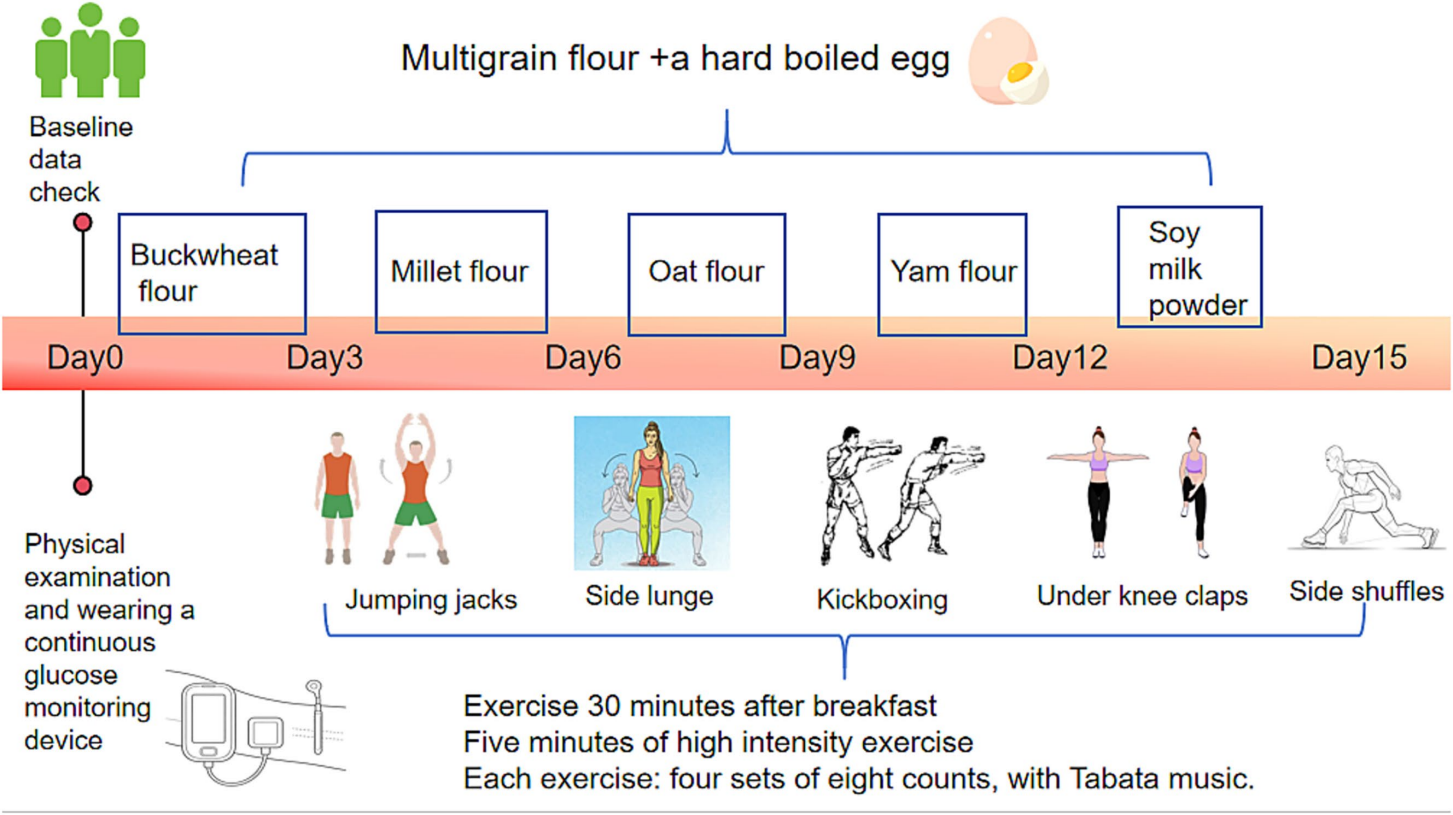
Experimental flowchart.

Furthermore, to evaluate systemic glucose and lipid metabolism, fasting venous blood samples were collected after an 8-h overnight fast at baseline (the day prior to the experiment) and post intervention (the day following the final exercise session). The serum biomarkers assessed included fasting blood glucose (FBG), total cholesterol (TC), triglycerides (TG), high-density lipoprotein cholesterol (HDL-C), and low- density lipoprotein cholesterol (LDL-C) (Figure 1).

### 3.3 Anthropometric measurements

To establish baseline characteristics, trained medical staff from the XX Normal University Hospital measured participants’ height, weight, and waist circumference between 8:00 and 9:00 a.m. on the 2 days prior to the intervention. Height and body weight were measured using an electronic stadiometer and scale. BMI was subsequently calculated as weight in kilograms divided by height in meters squared (kg/m^2^). During these measurements, participants were required to remove their shoes and heavy outer clothing and stand naturally at the center of the scale. In accordance with the Guidelines for Prevention and Control of Overweight and Obesity in Chinese Adults, overweight and obesity were defined as a BMI of ≥ 24.0 kg/m^2^ and ≥ 28.0 kg/m^2^, respectively (6). Waist circumference was assessed using a non-elastic tape measure. With participants standing naturally and their arms crossed over their chests, the horizontal circumference at the midpoint between the lower margin of the last palpable rib and the top of the iliac crest was measured to the nearest 0.1 cm. All anthropometric measurements were obtained in duplicate, and the average values were recorded for subsequent analysis.

### 3.4 Dietary assessment

Carbohydrates are the primary dietary determinant of postprandial glycemia, and foods with higher carbohydrate content elicit greater postprandial glucose peaks (28). Furthermore, the type of carbohydrate exerts varying effects on postprandial glycemic responses. Foods with a high glycemic index (GI) can cause sharp increases in postprandial glucose, whereas low GI foods induce a milder, gradual glycemic response that is conducive to maintaining stable blood glucose levels (29). The GI values of the five types of multigrain powder selected for this study—tartary buckwheat flour, millet flour, oat flour, yam flour, and soy milk powder—were obtained from the Chinese standard Labeling Specification for Glycemic Index of Prepackaged Foods (30) and the China Food Composition Tables, Standard Edition (6th Edition) (31). With the exception of millet powder, the multigrain powders selected for this study were classified as low-GI foods; specifically, the GI values were 48 for tartary buckwheat flour, 71 for millet flour, 50 for oat flour, 48 for yam flour, and 40 for soy milk powder. The GI of the accompanying boiled egg was approximately 0. Furthermore, none of the foods included in the test meals contained *trans* fatty acids. Considering that individuals with overweight or obesity often exhibit varying degrees of insulin resistance and impaired glucose metabolism, which may alter their glycemic regulatory capacity following dietary intake the degree of acute glycemic fluctuation elicited by a low-calorie breakfast in this population constituted the primary focus of this study (Table 1).

**Table 1:** Nutritional composition and glycemic index of the test meals.

Meal type	Total intake (g)	Total energy (kJ)	Total energy (kcal)	Total carbohydrate (g)	Total fat (g)	Total protein (g)	Total dietary fiber (g)
Tartary buckwheat flour + Boiled egg	70	592.6	141.6	13.54	5.30	8.90	1.16
Millet flour + Boiled egg	70	610.80	146.0	15.50	5.42	7.74	0.30
Oat flour + Boiled egg	70	580.20	138.7	9.28	5.92	9.00	1.60
Yam flour + Boiled egg	70	616.80	147.4	15.96	5.52	7.42	0.50
Soy milk powder + Boiled egg	70	668.00	159.7	2.80	9.68	13.50	2.20

### 3.5 Exercise protocol

This study aimed to examine the immediate effects of a single bout of short-duration moderate-to-vigorous exercise on postprandial glycemia under different multigrain powder interventions. Considering that the participants were predominantly middle-aged faculty and staff members, the exercise protocol was designed to feature simple, accessible, and space-efficient movements. The routine was developed with reference to commercial fitness applications (e.g., the Keep platform) and relevant studies on moderate-to-vigorous exercise regimens (32–36), and was guided by consultation with faculty experts from the Department of Physical Education at XX Normal University. The final routine consisted of five exercises—jumping jacks, lateral squats, shadow punches, under-leg clapping, and lateral shuffling—performed in time with Tabata-style music beats. Demonstration videos of the routine were pre-recorded by the research staff, who also provided instruction and supervised the exercise sessions. The protocol comprised alternating cycles of 40 s of moderate-to-vigorous exercise and 20 s of active recovery (stepping in place), totaling five rounds, to maximize exercise intensity within the limited time frame. Such brief rest intervals during exercise have been shown to attenuate glycemic responses (37).

During each exercise session, participants were equipped with a Polar heart rate monitor to measure their heart rates in real time. Research staff recorded the participants’ heart rates immediately after the completion of the 5-min exercise. Considering that excessive exercise intensity may increase the risk of adverse cardiovascular or musculoskeletal events in adults with overweight or obesity, heart rates maintained between 80% of maximal heart rate (HRmax) (38) were deemed appropriate for meeting the moderate-to-vigorous intensity requirements of this study. HRmax was calculated using the formula (220 – age) (39). During the experimental sessions, if a participant’s heart rate fell below the target zone, research staff instructed them to increase movement frequency or amplitude; conversely, if the heart rate exceeded the target zone, staff instructed them to transition to marching in place and reduce movement amplitude. The exercise session was immediately terminated if any participant experienced discomfort. The exercise protocol required the completion of five rounds per session, with a total of five sessions conducted throughout the experimental period. The overall exercise adherence rate was 87% (range: 75–100%).

### 3.6 Continuous glucose monitoring and postprandial response

Participants used a CGM system (Si-based Dynamic Pro, The First Hospital of Shanxi Medical University, Taiyuan, Shanxi, China) throughout the 15-day study period. The device has been approved by the National Medical Products Administration (NMPA) of China for clinical use. The sensor employs factory pre-calibration technology, eliminating the need for daily finger-prick blood calibration by participants during the wear period, thereby reducing user-induced experimental error. Clinical validation studies have demonstrated that the mean absolute relative difference (MARD) of this device is 8.83%, meeting international accuracy standards for clinical CGM devices (40). Research staff opened the packaging, disinfected the skin over the triceps brachii of the participant’s left or right upper arm with an alcohol swab, applied the sensor, and confirmed secure adhesion. The sensor was removed 2 h after the final meal of the 15-day intervention, followed by skin disinfection and cleansing (41). All aforementioned procedures were performed by the research staff.

After sensor application, participants were required to enter daily data into the companion smartphone application, including meal times, food types, and quantities for breakfast, lunch, and dinner, as well as exercise duration (in minutes) and sleep times. Interstitial glucose data were automatically recorded every 5 min. Outcome metrics provided by the continuous monitoring device included time in range (TIR), mean glucose, glucose standard deviation (SD), and coefficient of variation (CV) (38). Prior to monitoring, participants were trained to use the companion application. Research staff sent daily morning reminders for data entry, and participants were required to use the application to record daily lifestyle events. During the CGM monitoring period, participants maintained their habitual dietary and exercise habits, but were instructed to avoid strenuous exercise and alcohol consumption to minimize potential confounders of glycemic fluctuations.

Postprandial glycemic response data were extracted over a 2-h period following the consumption of the test breakfast. The multigrain powder was prepared by dissolving it in 200 mL of warm water and was consumed with one boiled egg. The breakfast was required to be consumed within 15 min. The two-hour postprandial glucose monitoring period commenced immediately upon the completion of breakfast. On the third day of each specific multigrain powder intervention phase, participants were required to perform 5 min of moderate-to-vigorous exercise. Data analysis was primarily based on the continuous glucose data obtained from the CGM system during this 2 h post breakfast period.

### 3.7 Outcome measures

To mitigate the risk of multiplicity bias and false positives, this study clearly delineated primary and secondary outcomes. The two-hour postprandial iAUC was defined as the sole primary outcome measure. Secondary outcomes included the total AUC, peak glucose increment, and GV. Additional exploratory parameters (e.g., mean glucose, time to peak, and ascending rate of glucose) were relegated to the Supplementary materials. Based on the continuous data obtained via CGM, the iAUC was calculated using the trapezoidal rule. This calculation employed the participants’ fasting interstitial glucose level as the baseline, including only the area above this baseline. A previous systematic review and meta-analysis of randomized controlled trials indicated that whole grain products can effectively reduce the incremental area under the glycemic curve in adults (42). As the most sensitive indicator of postprandial glycemic excursions, the iAUC was thus utilized to evaluate the acute glycemic effects of the interaction between multigrain powder consumption and postprandial exercise.

### 3.8 Secondary outcome measures

This study used the iAUC as the primary outcome measure to quantify the acute postprandial glycemic response elicited by multigrain powder consumption and its interaction with exercise. Additionally, several secondary outcome measures were included to comprehensively characterize the dynamics and variability of postprandial glycemic responses: postprandial peak glucose increment to assess the maximal magnitude of glucose elevation; GV to characterize glucose fluctuations; time to peak (TTP) to indirectly reflect gastric emptying rate; the ascending rate of blood glucose as a kinetic indicator of intestinal glucose absorption; and the AUC to evaluate overall postprandial glucose exposure. The selection of these indicators was based on substantial evidence from previous studies. Specifically, peak increment (43) and GV (44) have been shown to effectively reflect acute postprandial glycemic fluctuations; TTP and the ascending rate of blood glucose, as kinetic indicators, are associated with gastric emptying dynamics (45); and AUC is closely associated with insulin sensitivity and glucose absorption kinetics (46).

### 3.9 Sample size estimation and statistical analysis

Sample size estimation was performed using G*Power version 3.1 based on the study design. The significance level was set at *α* = 0.05, with a statistical power of 1 − *β* = 0.85 and an effect size of *f* = 0.25. The minimum required sample size was calculated to be 40 participants. To account for an anticipated 10% attrition rate and other potential sources of data loss, 50 overweight or obese adults were recruited. During the study, four participants withdrew because of health-related reasons, and three participants were unable to complete the follow-up assessment due to CGM sensor detachment. Ultimately, 43 participants (25 males and 18 females) completed the study and were included in the final analysis.

Statistical analyses were performed using Microsoft Excel 2016 and SPSS version 25.0, and figures were generated using GraphPad Prism version 10.0. The Shapiro–Wilk test was first applied to assess the normality of all 2-h PBG data. The results indicated that the data deviated significantly from a normal distribution (*p* < 0.05), and the data were right-skewed.

Considering that the data consisted of repeated measurements across different multigrain powder types and exercise conditions, and that the sample size was relatively small, the non-parametric Friedman test was employed to compare differences among the five multigrain powders, while the Wilcoxon signed rank test was used to evaluate glycemic changes related to exercise within each multigrain powder condition. Interaction effects were analyzed using GEE to assess the influence of multigrain powder type, postprandial exercise, and their interaction on postprandial glycemic parameters.

Postprandial blood glucose was set as the dependent variable. The GEE model included five multigrain powder types (Tartary buckwheat flour, Millet flour, Oat flour, Yam flour, Soy milk powder), postprandial exercise (two levels: with and without exercise), and their interaction as independent variables, with daily fasting blood glucose and sex incorporated as covariates to account for inter-individual differences in basal glucose metabolism. The working correlation matrix was specified as exchangeable. Given the non-normal distribution of the data, robust standard errors were applied for parameter estimation. When a main effect was statistically significant, estimated marginal means were calculated, followed by *post hoc*-pairwise comparisons with Bonferroni adjustment to correct for multiple testing. *p*-values < 0.05 were considered statistically significant.

## 4 Results

### 4.1 Baseline characteristics of participants

Baseline characteristics were assessed prior to the first intervention, including age, anthropometric measurements (height, weight, BMI, and waist circumference), and metabolic parameters (total cholesterol, triglycerides, high density lipoprotein, low density lipoprotein, and fasting blood glucose). Baseline characteristics of the 43 participants (25 males and 18 females) are presented as mean ± standard deviation (SD). The baseline characteristics of the study participants are summarized in Table 2.

**Table 2:** Baseline characteristics of participants.

Parameters	Total participants	Male participants	Female participants
*N*	43	25	18
Age (years)	43.65 ± 13.35	40.40 ± 13.35	48.17 ± 12.14
Height (m)	1.67 ± 0.08	1.73 ± 0.03	1.59 ± 0.03
Weight (kg)	73.83 ± 9.36	80.44 ± 5.82	64.65 ± 3.93
BMI(kg/m^2^)	26.25 ± 1.63	26.72 ± 1.56	25.59 ± 1.54
Waist circumference (cm)	85.83 ± 6.49	90.85 ± 2.90	78.87 ± 1.96
Total cholesterol (mmol/L)	4.86 ± 0.86	5.00 ± 0.86	4.65 ± 0.84
Triglycerides (mmol/L)	2.13 ± 1.80	2.51 ± 2.2	1.6 ± 0.83
High density lipoprotein (mmol/L)	1.25 ± 0.33	1.15 ± 0.2	1.38 ± 0.42
Low density lipoprotein (mmol/L)	3.52 ± 0.80	3.7 ± 0.72	3.26 ± 0.86
Glucose (mmol/L)	5.92 ± 1.32	6.11 ± 1.52	5.66 ± 0.95

### 4.2 Analysis of the effects of different multigrain interventions on two-hour postprandial blood glucose

To evaluate the overall effects of the five multigrain interventions, 2-h PBG was designated as the dependent variable, with the five intervention types (Tartary buckwheat flour, millet flour, oat flour, yam flour, and soy milk powder, coded as C1–C5 in the respective figures) serving as independent variables. Because the data across all groups failed to meet the assumption of normality, variables are expressed as medians (Q1, Q3). The Friedman test was employed for statistical analysis, with the significance level set at *α* = 0.05. The distribution of 2-h PBG for each group is presented in Table 3.

**Table 3:** Two hour postprandial blood glucose levels and Dunn’s *post hoc* comparison results of five multigrain flours.

Intervention group	Tartary buckwheat flour	Millet flour	Oat flour	Yam flour	Soy milk powder	2-h PBG (mmol/L)
Tartary buckwheat flour		0.0004^****^	0.0316^*^	<0.0001^****^	<0.0001^****^	6.87 (6.54, 7.22)
Millet flour			>0.9999^ns^	0.0002^****^	>0.9999^ns^	6.57 (6.14, 7.01)
Oat flour				<0.0001^****^	0.8924^ns^	6.62 (6.33, 7.20)
Yam flour					0.0017^**^	6.11 (6.01, 6.50)
Soy milk powder						6.44 (6.04, 6.59)

Data are expressed as median (Q1, Q3). ns: not significant (*P* > 0.05). *P < 0.05, **P < 0.01, ***P < 0.001, ****P < 0.0001.

**Figure 2:**
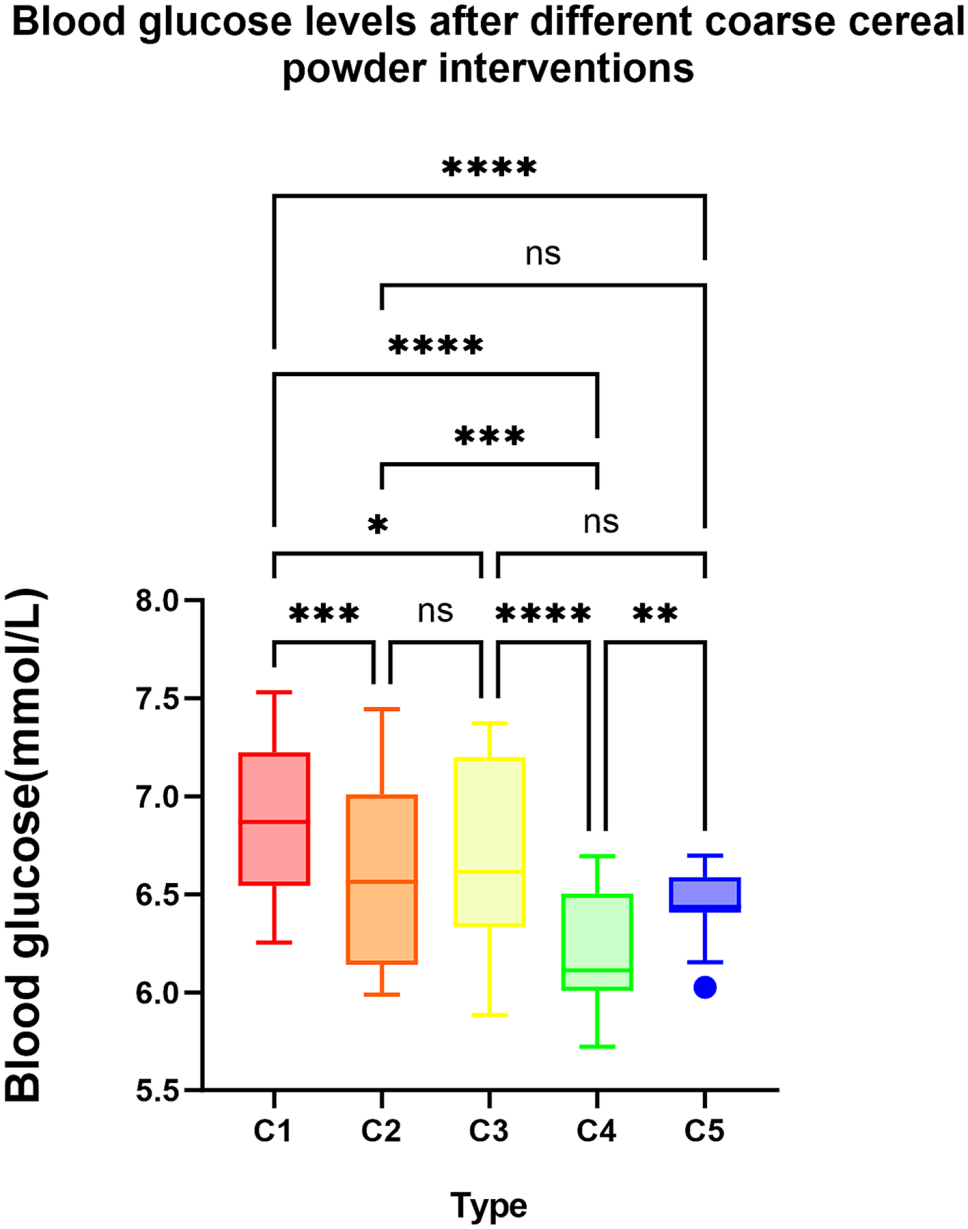
Effects of different multigrain interventions on 2-h PBG. C1–C5 represent Tartary buckwheat flour, millet flour, oat flour, yam flour, and soy milk powder, respectively. ns not significant (*p* > 0.05); * *p* < 0.05; ** *p* < 0.01; *** *p* < 0.001; ****p < 0.0001.

Significant differences in 2-h PBG were observed among the different intervention groups [χ^2^(4) = 73.92, *p* < 0.0001]. Dunn’s *post hoc*-comparisons (Table 3) revealed that the 2-h PBG in the Tartary buckwheat flour group was significantly higher than that in all other groups, whereas the level in the yam flour group was significantly lower. No statistically significant differences were observed among the remaining three groups, suggesting that millet flour, oat flour, and soy milk powder exerted comparable regulatory effects on 2-h PBG (Figure 2).

### 4.3 Effects of postprandial short duration moderate-to-vigorous intensity exercise on blood glucose

**Figure 3:**
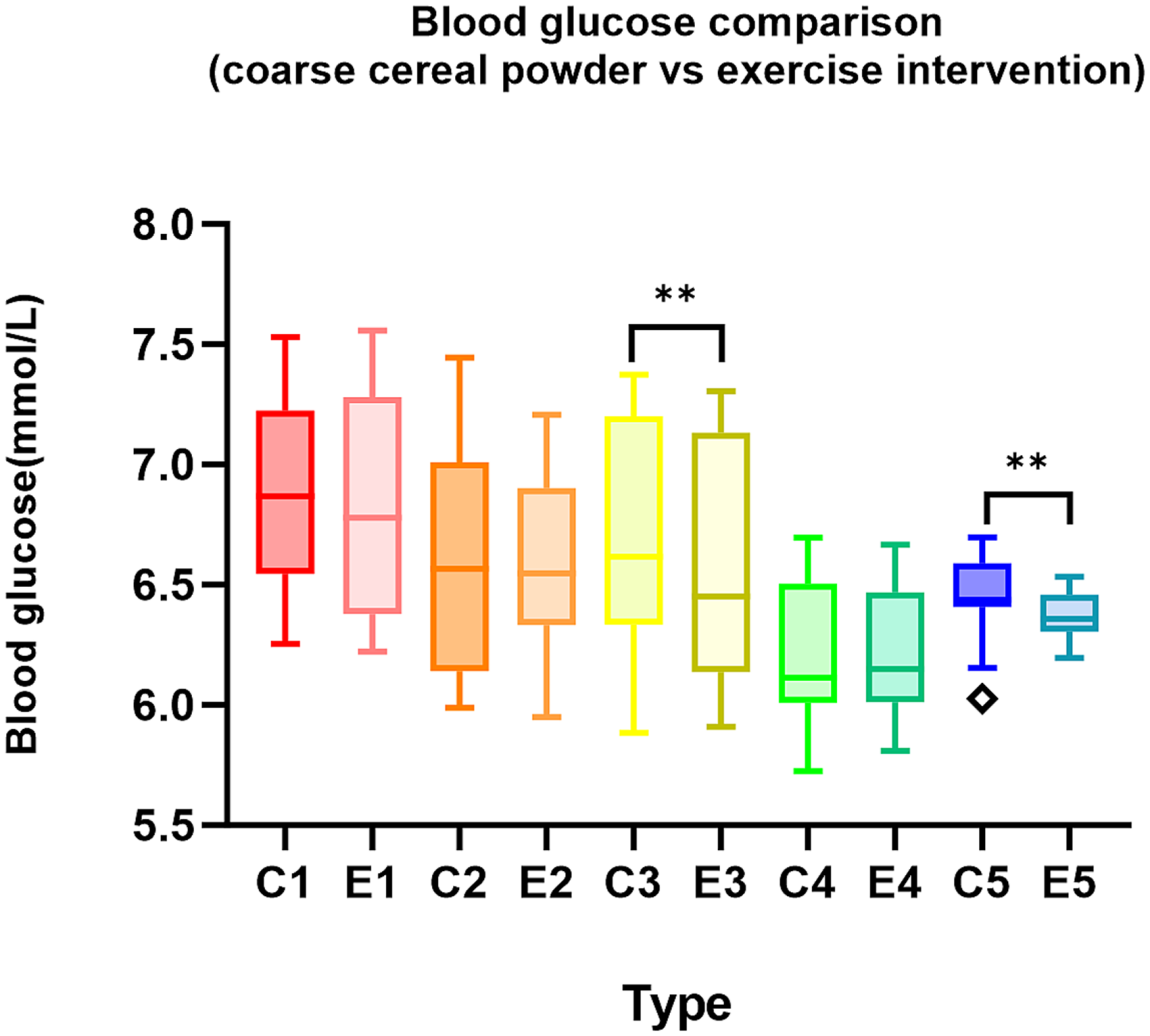
Effects of the exercise intervention on 2-h PBG across different multigrain interventions. C1–C5 represent tartary buckwheat flour, millet flour, oat flour, yam flour, and soy milk powder, respectively. E1–E5 represent the corresponding exercise groups for the tartary buckwheat flour, millet flour, oat flour, yam flour, and soy milk powder interventions, respectively, **p < 0.01.

Because the 2-h PBG data did not satisfy the assumption of normality, the Wilcoxon signed-rank test was employed to evaluate the overall effect of the exercise intervention on 2-h PBG and to compare the differences in 2-h PBG before and after the intervention across the different multigrain interventions. The results demonstrated that the exercise intervention significantly reduced 2-h PBG levels in the oat flour (*p* = 0.0057) and soy milk powder (*p* = 0.0017) groups, but had no significant effect in the tartary buckwheat flour, millet flour, and yam flour groups (*p* > 0.05). Furthermore, comparisons of the changes in 2-h PBG before and after the exercise intervention among the groups revealed no statistical significance (*p* > 0.05). Under the different multigrain dietary backgrounds in this study, short-duration moderate-to-vigorous intensity exercise did not demonstrate a universally significant glucose-lowering effect, and the type of multigrain intervention did not exert a significant moderating effect on the glucose-lowering action of the exercise (Figure 3).

### 4.4 Primary outcome measures

To evaluate the regulatory effects of the different multigrain interventions combined with the exercise intervention on the 2-h PBG this study used baseline blood glucose as a covariate and applied a GEE model to analyze the main effects of the multigrain intervention type, exercise, and their interaction. The results indicated that the main effect of the multigrain intervention type was significant [Wald χ^2^(4) = 64.997, *p* < 0.001], whereas the main effect of the exercise intervention [Wald χ^2^(1) = 0.197, *p* = 0.657] and the interaction effect between the multigrain intervention and exercise [Wald χ^2^(4) = 0.594, *p* = 0.964] showed no statistical significance. This finding suggests that the variation in the 2-h PBG iAUC among participants was primarily influenced by the independent effect of the multigrain intervention type.

Differences were observed in the estimated marginal means of the2-h PBG AUC among the five multigrain intervention groups (Table 4). The GV was lowest in the soy milk powder group (0.068 ± 0.004, 95% CI: 0.060–0.076) and highest in the tartary buckwheat flour group (0.123 ± 0.006, 95% CI: 0.112–0.134). Bonferroni pairwise comparisons (Table 5) revealed significant differences in iAUC levels across the different multigrain interventions. The estimated marginal mean of the iAUC was lowest in the soy milk powder group (51.494 ± 6.335), which was significantly lower than that in the oat flour group (mean difference: −55.927, *p* < 0.01), the millet flour group (mean difference: −43.825*, p* < 0.01), and the Tartary buckwheat flour group (mean difference: −42.048, *p* < 0.01). The iAUC in the yam flour group (71.763 ± 7.722) was also significantly lower than that in the oat flour group (mean difference: −35.658, *p* < 0.05). No statistically significant differences in iAUC were observed among the millet flour, tartary buckwheat flour, and oat flour groups, or between the yam flour and soy milk powder groups (*p* > 0.05).

**Table 4:** Estimated marginal means of the 2-h PBG incremental area under the curve across different multigrain interventions ((mmol/L)·min).

Group (*n* = 5)	Mean ± SE	95% CI	
		Lower bound	Upper bound
Tartary buckwheat flour	93.542 ± 8.031	77.801	109.283
Millet flour	95.319 ± 10.078	75.567	115.071
Oat flour	107.421 ± 10.591	86.662	128.179
Yam flour	71.763 ± 7.722	56.627	86.899
Soy milk powder	51.494 ± 6.335	39.078	63.911

**Table 5:** Pairwise comparisons of differences in blood glucose incremental area under the curve among different multigrain groups ((mmol/L)·min).

Group (*n* = 5)	Tartary buckwheat flour	Millet flour	Oat flour	Yam flour
Millet flour	1.777	–	–	–
Oat flour	13.879	12.102	–	–
Yam flour	−21.779	−23.556	−35.658^*^	-
Soy milk powder	−42.048^**^	−43.825^**^	−55.927^**^	−20.269

*P < 0.05, **P < 0.01.

### 4.5 Secondary outcome measures

#### 4.5.1 Postprandial peak glucose increment

Regarding the regulatory effects of the different multigrain interventions combined with exercise on the 2 h postprandial peak glucose increment, the main effect of the multigrain intervention type was significant [Wald χ^2^(4) = 45.989, *p* < 0.001]; the main effect of exercise was not statistically significant [Wald χ^2^(1) = 0.031, *p* = 0.860]; and the interaction effect between the multigrain intervention and exercise was not statistically significant [Wald χ^2^(4) = 1.199, *p* = 0.878]. Differences were observed in the estimated marginal means of the two-hour postprandial peak glucose increment among the five multigrain intervention groups (Table 6). The peak glucose increment was lowest in the soy milk powder group (1.194 ± 0.101, 95% CI: 0.997–1.391) and highest in the tartary buckwheat flour group (2.074 ± 0.149, 95% CI: 1.782–2.365). Bonferroni *post hoc*-pairwise comparisons (Table 7) revealed significant differences in postprandial peak glucose increment levels across the different multigrain interventions. The peak glucose increment in the soy milk powder group was significantly lower than that in the oat flour group (mean difference: −0.880, *p* < 0.001), the Tartary buckwheat flour group (mean difference: −0.811, *p* < 0.001), and the millet flour group (mean difference: −0.763, *p* = 0.001). The difference in the peak glucose increment between the soy milk powder group and the yam flour group approached statistical significance (mean difference: −0.479, *p* = 0.057). No statistically significant differences in the peak glucose increment were observed among the tartary buckwheat flour, millet flour, oat flour, and yam flour groups in pairwise comparisons (*p* > 0.05). These findings indicate that soy milk powder demonstrated an acute response in reducing the 2 h postprandial peak glucose increment.

**Table 6:** Comparison of estimated marginal means of the two-hour postprandial peak glucose increment across different multigrain intervention groups (mmol/L).

Group (*n* = 5)	Mean ± SE	95% CI	
		Lower bound	Upper bound
Tartary buckwheat flour	2.004 ± 0.136	1.737	2.271
Millet flour	1.957 ± 0.162	1.640	2.274
Oat flour	2.074 ± 0.149	1.782	2.365
Yam flour	1.672 ± 0.139	1.400	1.944
Soy milk powder	1.194 ± 0.101	0.997	1.391

**Table 7:** Pairwise comparisons of differences in the peak glucose increment among different multigrain groups (mmol/L).

Group (*n* = 5)	Tartary buckwheat flour	Millet flour	Oat flour	Yam flour
Millet flour	−0.047	-	-	-
Oat flour	0.070	0.117	-	-
Yam flour	−0.332	−0.285	−0.402^*^	-
Soy milk powder	−0.811^**^	−0.763^**^	−0.880^**^	−0.479

**P < 0.01, *p < 0.05.

#### 4.5.2 Glycemic variability

Regarding GV, the main effect of the multigrain intervention type was statistically significant (Wald χ^2^(4) = 86.352, *p* < 0.001); the main effect of exercise was not statistically significant (Wald χ^2^(1) = 0.015, *p* = 0.903); and the interaction effect between the multigrain intervention and exercise was not statistically significant (Wald χ^2^(4) = 0.798, *p* = 0.939). Under the conditions of this study, different types of multigrain interventions were confirmed to affect the GV of the 2-h PBG. Neither exercise nor the interaction between exercise and diet showed significant effects within this experimental context.

Differences were observed in the estimated marginal means of two-hour postprandial GV among the five multigrain intervention groups (Table 8). The GV was lowest in the soy milk powder group (0.068 ± 0.004, 95% CI: 0.060–0.076) and highest in the tartary buckwheat flour group (0.123 ± 0.006, 95% CI: 0.112–0.134). Pairwise comparisons (Table 9) revealed that the GV in the soy milk powder group was significantly lower than that in the tartary buckwheat flour group (mean difference: −0.054, *p* < 0.01), the oat flour group (mean difference: −0.048, *p* < 0.01), the millet flour group (mean difference: −0.046, *p* < 0.01), and the yam flour group (mean difference: −0.032, *p* < 0.01). The GV in the yam flour group was significantly lower than that in the tartary buckwheat flour group (mean difference: −0.023, *p* < 0.05), whereas the differences between the yam flour group and both the oat flour and millet flour groups did not reach statistical significance (*p* > 0.05). No statistically significant differences in GV were observed among the tartary buckwheat flour, millet flour, and oat flour groups in pairwise comparisons. These findings demonstrate that, among the multigrain interventions selected in this study, soy milk powder could improve the acute response of two-hour postprandial GV; the acute response effect of yam flour on improving two-hour postprandial GV was inferior to that of soy milk powder; and the postprandial GV induced by the tartary buckwheat flour, millet flour, and oat flour interventions was relatively large, with no significant differences among the three.

**Table 8:** Comparison of estimated marginal means of two-hour postprandial GV across different multigrain intervention groups (%).

Group (*n* = 5)	Mean ± SE	95% CI	
		Lower bound	Upper bound
Tartary buckwheat flour	0.123 ± 0.006	0.112	0.134
Millet flour	0.114 ± 0.006	0.103	0.125
Oat flour	0.116 ± 0.006	0.104	0.129
Yam flour	0.100 ± 0.006	0.088	0.112
Soy milk powder	0.068 ± 0.004	0.060	0.076

**Table 9:** Pairwise comparisons of differences in GV among different multigrain groups (%).

Group (*n* = 5)	Tartary buckwheat flour	Millet flour	Oat flour	Yam flour
Millet flour	−0.009	–	–	–
Oat flour	−0.006	0.002	–	–
Yam flour	−0.023^*^	−0.014	−0.016	–
Soy milk powder	−0.054^**^	−0.046^**^	−0.048^**^	−0.032^**^

*P < 0.05, **P < 0.01.

#### 4.5.3 Time to peak

A GEE model was applied to analyze the effects of the multigrain interventions, exercise, and their interaction on the TTP of 2-h PBG. The results indicated that the interaction effect between the multigrain intervention and exercise was not significant [Wald χ^2^(4) = 3.653, *p* = 0.455]; neither the main effect of the multigrain intervention type [Wald χ^2^(4) = 6.138, *p* = 0.189] nor the main effect of exercise [Wald χ^2^(1) = 0.691, *p* = 0.406] achieved statistical significance. Under the experimental conditions designed in this study, neither the multigrain interventions nor exercise induced significant differences in the TTP of 2-h PBG.

#### 4.5.4 Ascending rate of blood glucose

With respect to the ascending rate of blood glucose during the two-hour postprandial period, the interaction effect between the multigrain intervention and exercise was not statistically significant [Wald χ^2^(4) = 2.321, *p* = 0.677]; the main effect of the multigrain intervention type did not reach statistical significance but demonstrated a marginally significant trend [Wald χ^2^(4) = 8.910, *p* = 0.063]; and the main effect of exercise was not statistically significant [Wald χ^2^(1) = 0.003, *p* = 0.955]. The different types of multigrain interventions and exercise intervention protocols designed in this study did not produce significant effects on the ascending rate of 2-h PBG.

#### 4.5.5 Area under the curve

Regarding the AUC, the GEE model indicated that the main effect of the multigrain intervention type was statistically significant [Wald χ^2^(4) = 25.373, *p* < 0.001]; the main effect of exercise was not statistically significant [Wald χ^2^(1) = 0.001, *p* = 0.975]; and the interaction effect between the multigrain intervention and exercise was not significant [Wald χ^2^(4) = 1.326, *p* = 0.857]. Differences were observed in the estimated marginal means of the 2-h PBG AUC among the five multigrain intervention groups (Table 10). The mean AUC was lowest in the yam flour group (746.773 ± 13.669, 95% CI: 719.982–773.563) and highest in the tartary buckwheat flour group (825.693 ± 23.934, 95% CI: 778.783–872.604). Pairwise comparisons (Table 11) revealed that the AUC in the yam flour group was significantly lower than that in the tartary buckwheat flour group (mean difference: −78.921, *p* < 0.01), the millet flour group (mean difference: −46.032, *p* < 0.05), and the oat flour group (mean difference: −53.720, *p* < 0.01). In addition, the AUC in the soy milk powder group was also significantly lower than that in the tartary buckwheat flour group (mean difference: −55.099, *p* < 0.05). No other pairwise comparisons reached statistical significance. Under the experimental conditions of this study, yam flour demonstrated the strongest effect in reducing the 2-h PBG AUC.

**Table 10:** Comparison of estimated marginal means of the 2-h PBG AUC across different multigrain intervention groups ((mmol/L)·min).

Group (*n* = 5)	Mean ± SE	95% CI	
		Lower bound	Upper bound
Millet flour	825.693 ± 23.934	778.783	872.604
Oat flour	792.805 ± 17.502	758.501	827.108
Yam flour	800.493 ± 18.774	763.696	837.289
Soy milk powder	746.773 ± 13.669	719.982	773.563
Millet flour	770.594 ± 16.152	738.936	802.253

**Table 11:** Pairwise comparisons of differences in the AUC among different multigrain groups [(mmol/L)·min].

Group (*n* = 5)	Tartary buckwheat flour	Millet flour	Oat flour	Yam flour
Millet flour	−32.889	-	-	-
Oat flour	−25.201	7.688	-	-
Yam flour	−78.921^**^	−46.032^*^	−53.720^**^	-
Soy milk powder	−55.099^*^	−22.210	−29.898	23.822

*P < 0.05, **P < 0.01.

### 4.6 Summary

This study included 43 overweight and obese adults. Baseline characteristics indicated a mean BMI of 26.25 kg/m^2^. Total cholesterol, triglyceride, and low density lipoprotein levels were at the upper limits of the normal range, whereas high-density lipoprotein levels were at the lower limit. These baseline data suggest that participants exhibited mild metabolic dysfunction.

The effects of five fixed-sequence whole grain flours combined with exercise on 2-h PBG were evaluated as follows. Different whole grain flours significantly influenced 2-h PBG glucose. The tartary buckwheat flour group demonstrated the highest blood glucose, while the yam flour group showed the lowest. Millet flour, oat flour, and soybean milk powder produced comparable effects. These differences may be attributed to variations in the nutritional composition of the flours. Acute, single session, 5 min moderate-to-vigorous intensity exercise performed postprandially significantly reduced blood glucose following ingestion of oat flour and soybean milk powder. Exercise had no significant effect on the other flours, and the interaction between exercise and flour type was not significant.

Analysis of the primary outcome revealed that the iAUC was mainly determined by flour type. The iAUC for soybean milk flour was significantly lower than that for oat flour, millet flour, and tartary buckwheat flour, whereas the iAUC for yam flour was lower than that for oat flour. Secondary outcomes showed that blood glucose peak increment, CV, and total AUC varied according to flour type. Soybean milk powder demonstrated the lowest peak increment and CV, and yam flour showed greater efficacy than the other flours in reducing AUC. Exercise did not exert statistically significant effects on either primary or secondary outcomes.

Overall, flour type exerted a stronger influence on and 2-h PBG glycemic variability than exercise. The study used a fixed sequence design without washout periods between flours and applied fixed doses of flours, which led to variations in nutritional composition. These factors may have affected the magnitude of the observed effects.

## 5 Discussion

This study examined the acute effects of five different multigrain interventions combined with short duration moderate-to-vigorous intensity exercise on 2-h PBG. The results demonstrated that, under the experimental conditions of this study, the type of multigrain intervention consumed exerted a significant and independent effect on the regulation of 2-h PBG. Soy milk powder and yam flour demonstrated certain advantages in modulating postprandial blood glucose levels. Soy milk powder was more effective in reducing the iAUC, peak increment, and GV of 2-h PBG, whereas yam flour achieved a greater improvement in reducing the total AUC. These differential effects may reflect the distinct nutritional profiles and physiological mechanisms associated with the different types of multigrain interventions. Due to the absence of an isocaloric intervention design in this study, the five whole grain flours varied in carbohydrate, fat, protein, and dietary fiber content and composition. These variations may have contributed to the observed differences in postprandial glycemic responses. Soybean milk powder contained a relatively high protein content, particularly plant derived proteins. Yam flour was rich in carbohydrates and resistant starch. Oat flour was abundant in *β*-glucan. Tartary buckwheat flour contained higher carbohydrate levels along with rutin and resistant starch. Millet flour was rich in dietary fiber (47–51).

Higher protein content can delay gastric emptying and reduce the rate of carbohydrate absorption, while resistant starch is less susceptible to enzymatic degradation in the digestive tract, thereby slowing carbohydrate absorption and attenuating postprandial glucose elevation. These nutritional differences may act as potential confounders affecting glycemic responses and should be considered when interpreting the results. Therefore, future studies should adopt isocaloric conditions to independently examine and elucidate the interactive effects of individual nutritional components.

Given that the test meal dose in this study was relatively small and did not represent a standard breakfast for most individuals, it may serve as a reference for high-calorie breakfast substitutions; however, this study is primarily intended to provide strategic insights into blood glucose management using meal replacement foods for overweight and obese populations. The test meals in this study exhibited immediate and significant differences in 2-h PBG, which may be attributable to the rich content of soy isoflavones, soy oligosaccharides, and phytosterols in the soy milk powder. These components can stimulate gastrointestinal hormones to delay gastric emptying, reduce the initial intestinal glucose flux, lower glucose peaks, and decrease the glycemic iAUC. Specifically, proteins and isoflavones can amplify incretin responses, such as glucagon-like peptide-1 and glucose dependent insulinotropic polypeptide improve the early-phase insulin response, flatten the glycemic curve, and reduce the glycemic index, peak increment, and GV, thereby improving early postprandial glycemic control (52).

In contrast, yam flour is rich in resistant starch, polysaccharides, and dietary fiber, which can form a physical barrier to slow carbohydrate digestion and delay intestinal glucose absorption. Moreover, yam polysaccharides activate the AMP activated protein kinase signaling (AMPK) pathway to enhance glucose uptake efficiency in muscle cells, and polyphenols in yam protect pancreatic *β* cells and promote insulin secretion, thereby reducing the total amount of available glucose at 2 h post-breakfast and lowering overall glycemic exposure (53–55). However, because this study did not measure insulin secretion following each multigrain intervention or the biomarkers involved in the mechanistic pathways activated by 2-h PBG, the long-term effects of soy milk powder and yam flour on daily glycemic control require further validation using additional biomarker outcome measures.

The short duration (5 min) moderate-to-vigorous intensity exercise performed 30 min post-prandially did not produce a significant overall glucose-lowering effect. These results support Hypothesis 1 of this study, indicating that different types of multigrain interventions induce distinct postprandial glycemic responses. These findings are consistent with previous studies regarding the effects of multigrain interventions on postprandial blood glucose (56, 57). Prior reviews have concluded that performing 2–5 min of light walking or simple body-weight resistance activities at 30 min intervals after meals can effectively alleviate postprandial glycemic fluctuations. The present study focused on whether a single bout of a 5 min simple fitness exercise performed postprandially could attenuate glycemic fluctuations (15). However, our results indicated that a single bout of 5-min moderate-to-vigorous intensity exercise did not induce significant changes in glycemic-related characteristics among the participants. This lack of efficacy may be attributable to the short exercise duration, which was likely insufficient to fully activate skeletal muscle glucose transport pathways and the AMPK signaling pathway, thereby failing to trigger GLUT4 translocation to the cell membrane. This study only observed acute changes in 2-h PBG following a single short duration intervention, without incorporating various time points, exercise modalities, or intensities, nor exploring the cumulative effects of long-term multigrain interventions combined with short-duration exercise. Furthermore, the relatively small sample size may have amplified the influence of substantial individual variation on the immediate effects of the short-duration exercise, highlighting a critical area for expansion in future research.

Due to budget constraints, this study did not evaluate different doses of the multigrain interventions or analyze the acute glycemic effects of interventions with varying dietary fiber contents. Therefore, future research should design exercise protocols with diverse modalities, intensities, and durations, focusing on the synergy between exercise and nutrition to explore personalized diet-exercise regimens. Such regimens would be suitable for early intervention in blood glucose management among overweight or obese adults, ultimately reducing the risk of progression to diabetes. In conclusion, the evidence indicates that different types of multigrain interventions can affect distinct characteristics of postprandial blood glucose, although the glycemic fluctuation responses triggered at different time points may vary. Future studies should expand the sample size and establish long term follow-up comparisons incorporating various exercise protocols alongside distinct multigrain interventions. This approach will facilitate the exploration of specific exercise regimens tailored to diverse dietary contexts, thereby providing precise diet and exercise strategies for optimal glycemic regulation.

This study employed a within-subject design to reduce the influence of inter-individual differences on glycemic changes, with a particular focus on glycemic variations in overweight or obese adults under the interaction of the multigrain interventions and exercise. However, because the recruited cohort exclusively comprised faculty and staff members with a recruitment rate of 58.2%, this study has several limitations. First, this study did not account for potential sex differences. Due to the relatively small sample size and the uneven distribution of male and female participants, it was not possible to adequately assess sex-specific responses. Future studies should recruit more female participants to better investigate and explore potential sex-specific effects. Second, this study only focused on the acute effects of short-duration exercise and was unable to evaluate the long-term effects of multiple accumulated short-duration bouts on blood glucose levels. Future studies should design short-duration exercise protocols with varying frequencies to investigate the chronic effects of regular exercise on postprandial glycemic responses. Finally, although every effort was made to provide standardized guidance regarding diet and exercise, some unforeseen life events may have occurred as confounding factors affecting the assessment of the acute glycemic effects. Moreover, no washout period was incorporated during the dietary and exercise intervention phases. Although the focus of this study was on immediate changes and acute effects, potential carryover effects during the testing phases could not be entirely excluded. Future studies should incorporate adequate washout periods and rigorously control for extraneous variables to reduce potential confounding effects on blood glucose.

## Data Availability

The raw data supporting the conclusions of this article will be made available by the corresponding author upon request.
