# Supplementary material for: A study on the effects of a whole grain diet combined with short duration exercise on postprandial glucose levels in overweight and obese adults
Source: Front Nutr. 2026 Jul 1;13:1886129. doi: 10.3389/fnut.2026.1886129 (PMC13369248; doi:10.3389/fnut.2026.1886129)
Supplement: Supplementary file 1 [file Supplementary_file_1.DOCX]

### ****Supplementary Figures****


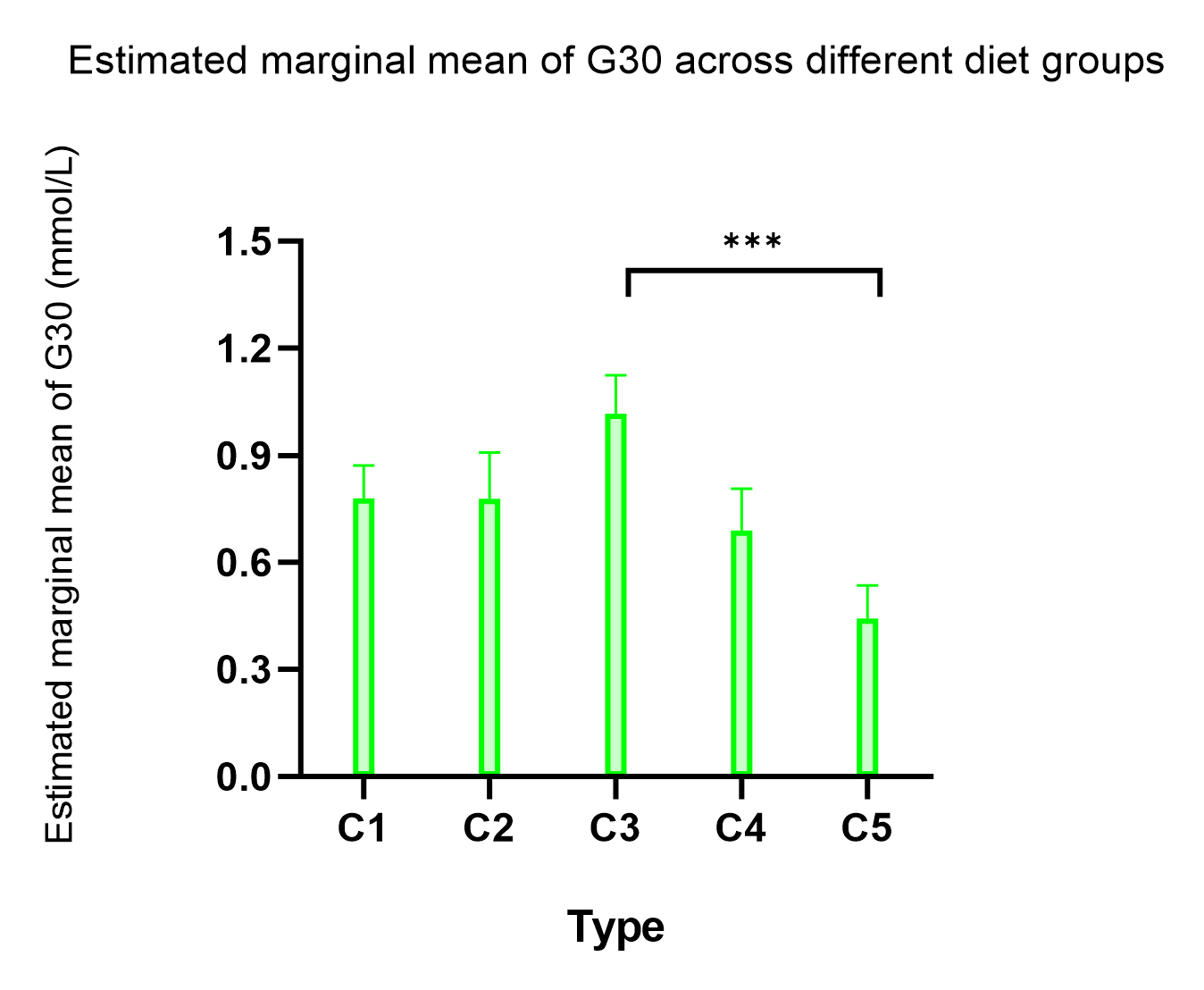


Figure 1Comparison of estimated marginal means for G30 across different whole grain flour groups

Figure caption: C1—C5 represent buckwheat flour, millet flour, oat flour,yam flour, and soy milk powder, respectively

***: *P* < 0.001


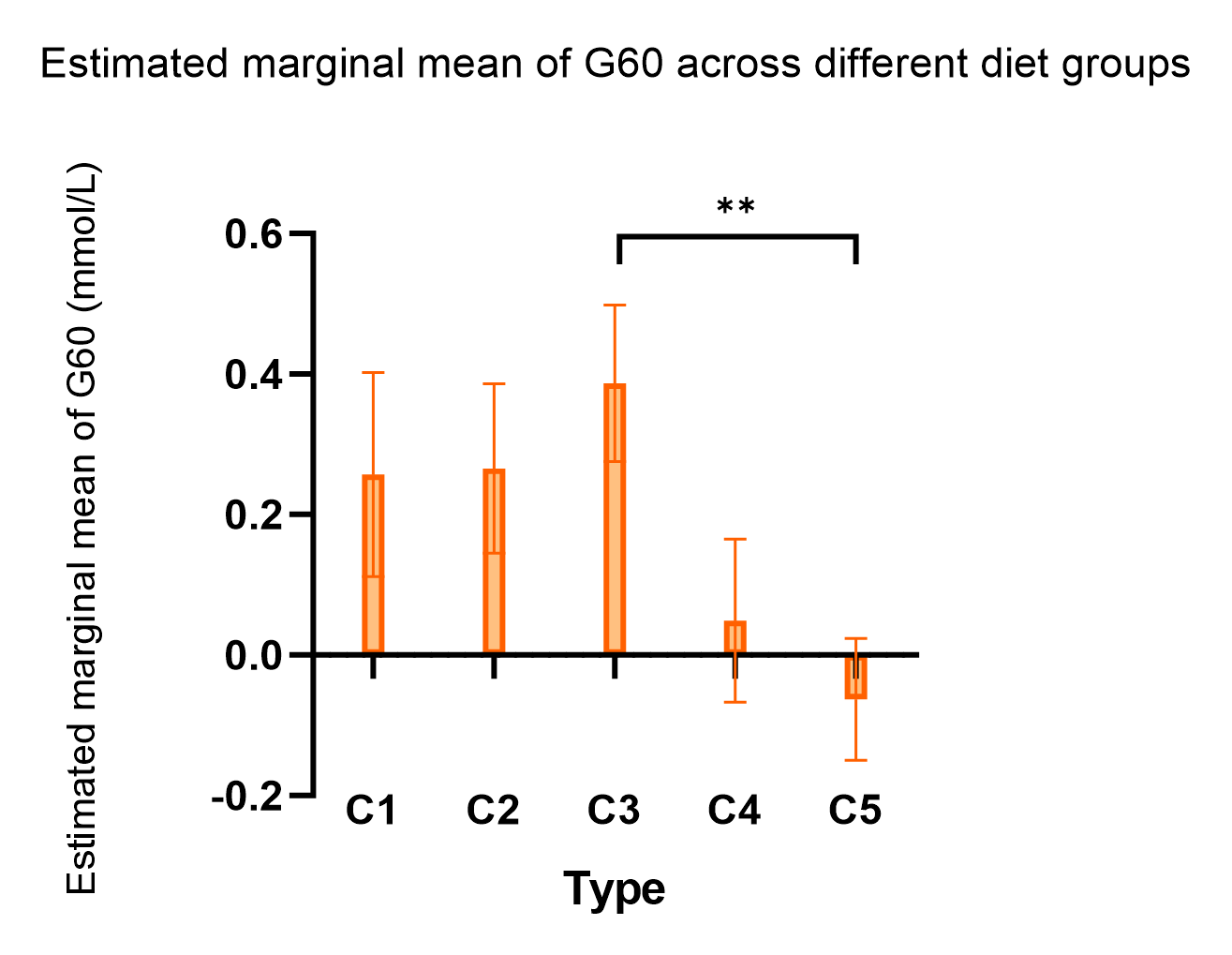


Figure 2 Comparison of estimated marginal means for G60 across different whole grain flour groups

Figure caption: C1—C5 represent buckwheat flour, millet flour, oat flour,yam flour, and soy milk powder, respectively

**:*P*< 0.01


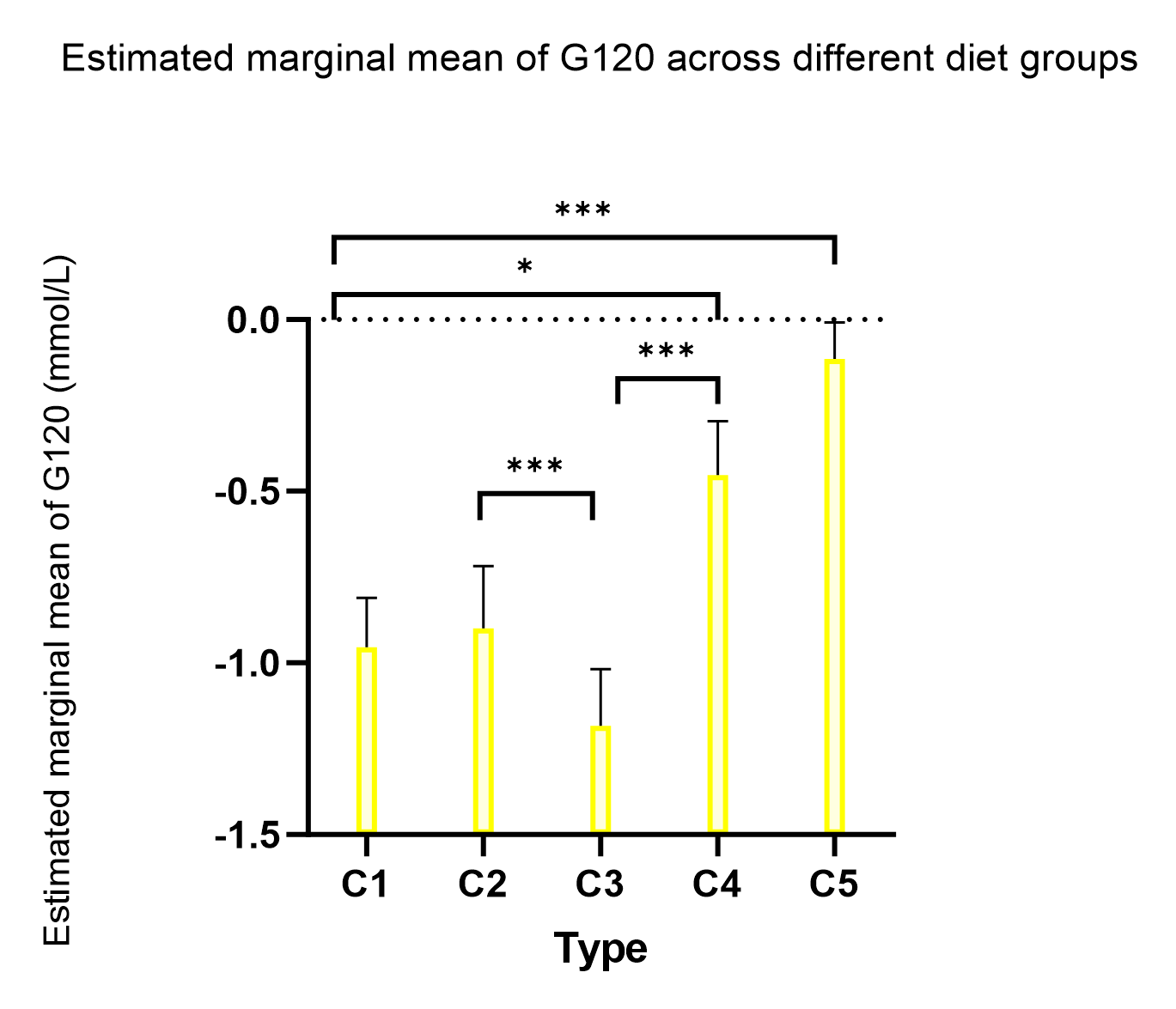


Figure 3 Comparison of estimated marginal means for G120 across different whole grain flour groups

Figure caption: C1—C5 represent buckwheat flour, millet flour, oat flour,yam flour, and soy milk powder, respectively

*: *P* < 0.05; ***:*P*< 0.001


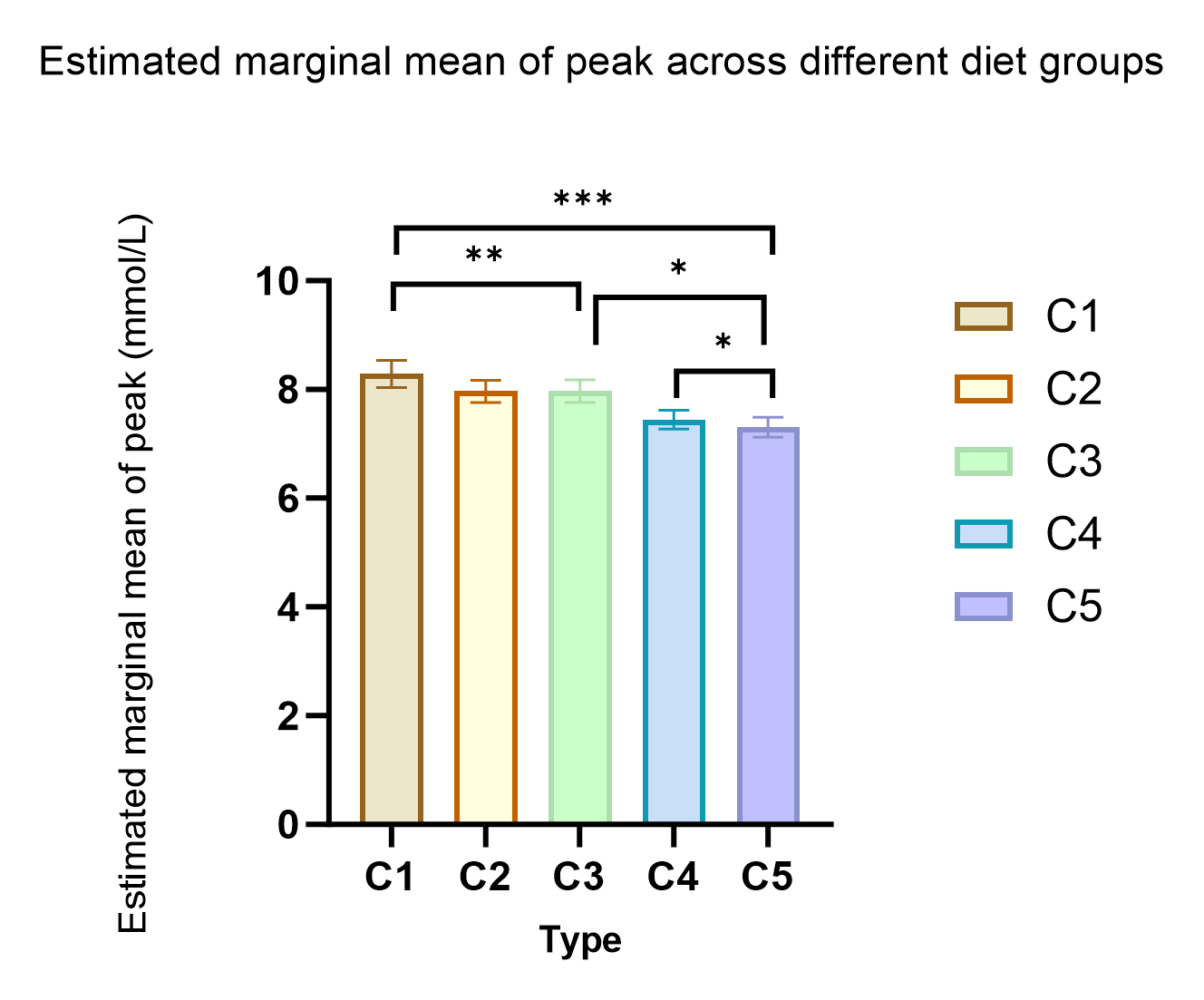


Figure 4 Comparison of estimated mean changes in postprandial blood glucose peaks two hours after meals for different whole grain flour groups

Figure caption: C1—C5 represent buckwheat flour, millet flour, oat flour,yam flour, and soy milk powder, respectively

*:*P* < 0.05, **: *P*< 0.01, ***: *P* < 0.001,


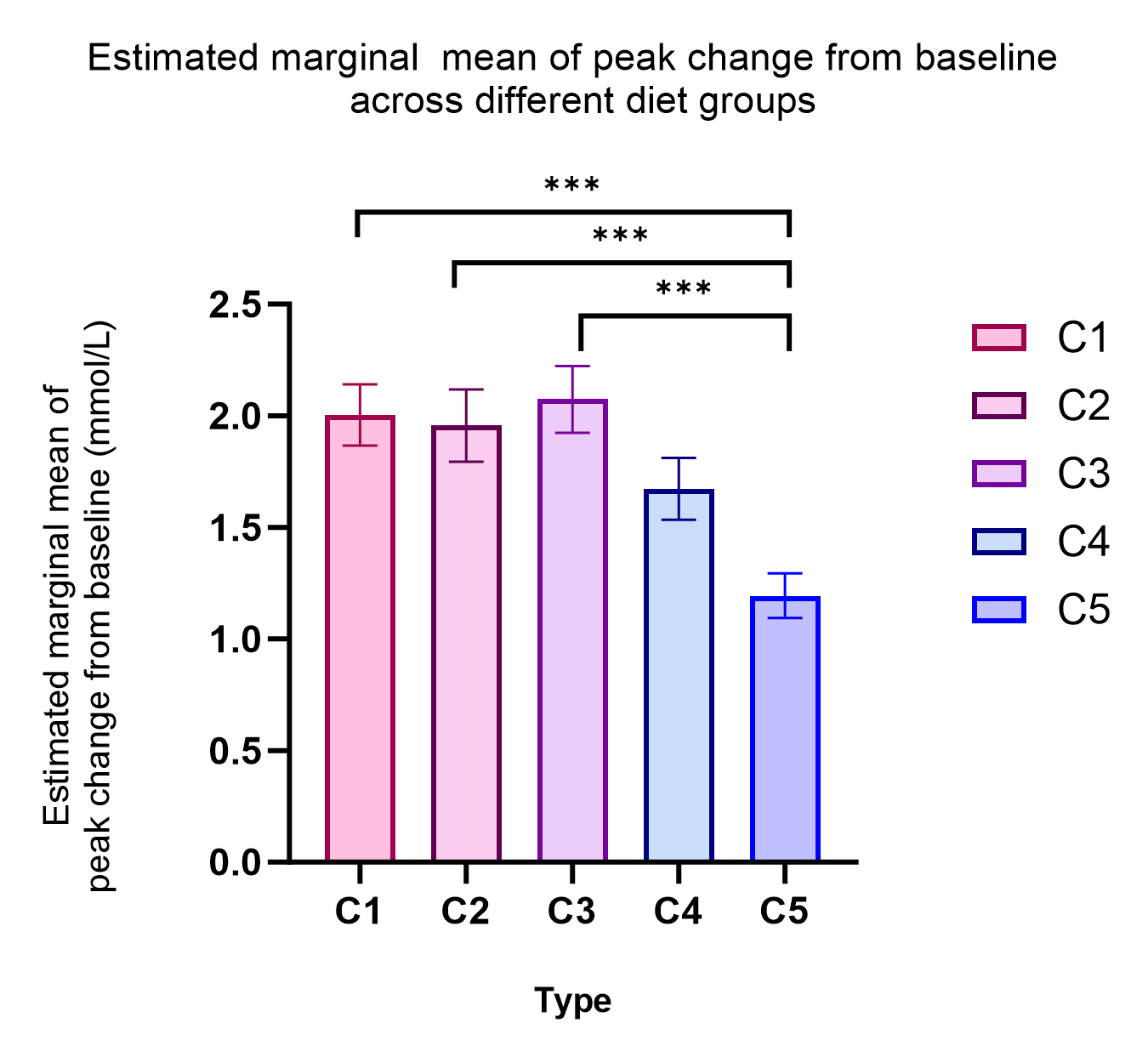


Figure 5 Comparison of estimated mean marginal increases in postprandial blood glucose peaks for different whole grain flour groups

Figure caption: C1—C5 represent buckwheat flour, millet flour, oat flour,yam flour, and soy milk powder, respectively

***: *P* < 0.001


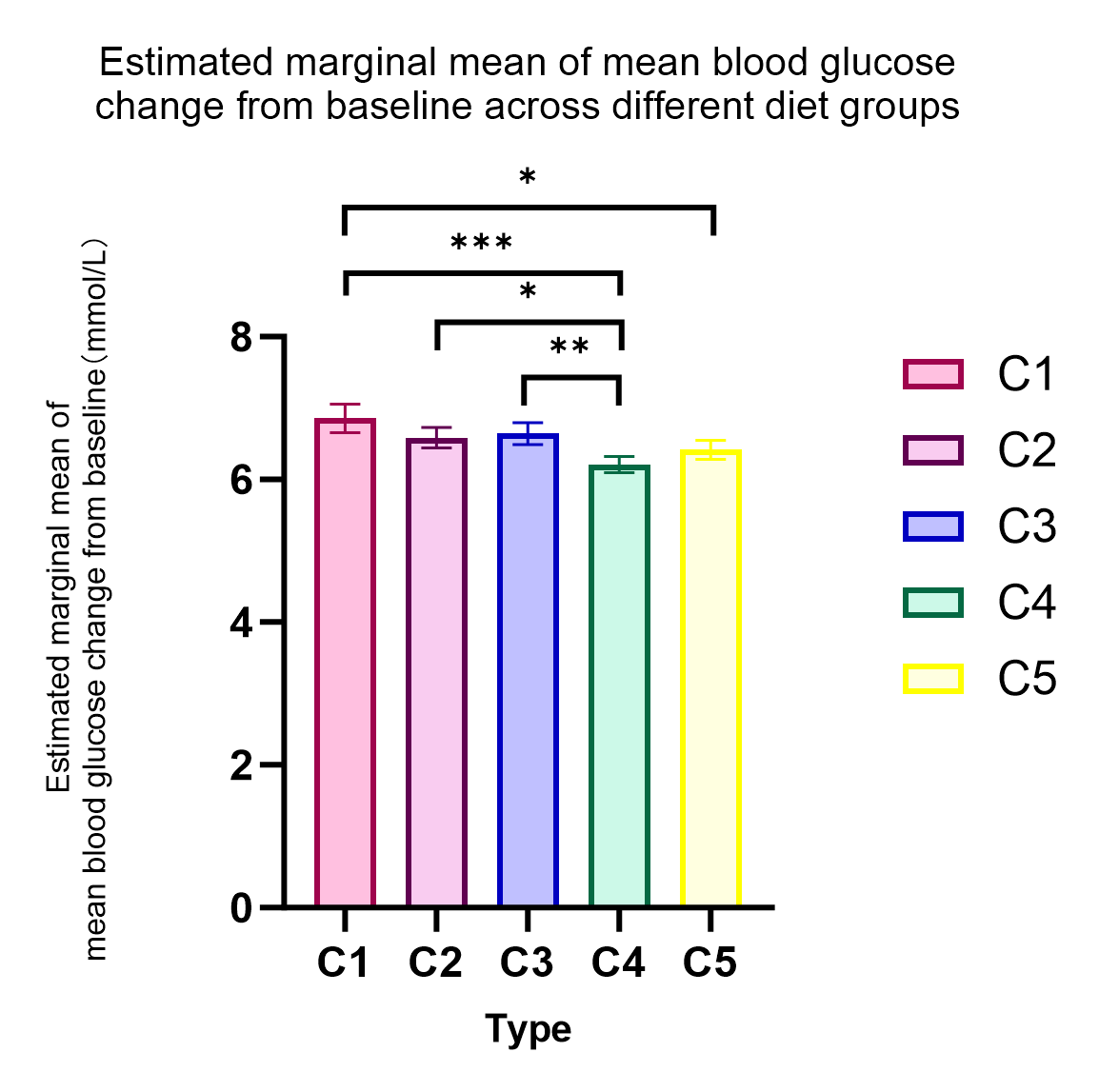


Figure 6 Comparison of estimated marginal means for mean blood glucose levels two hours after meals across different whole grain flour groups

Figure caption: C1—C5 represent buckwheat flour, millet flour, oat flour,yam flour, and soy milk powder, respectively

*: *P* < 0.05; **:*P* < 0.01***:*P* < 0.001


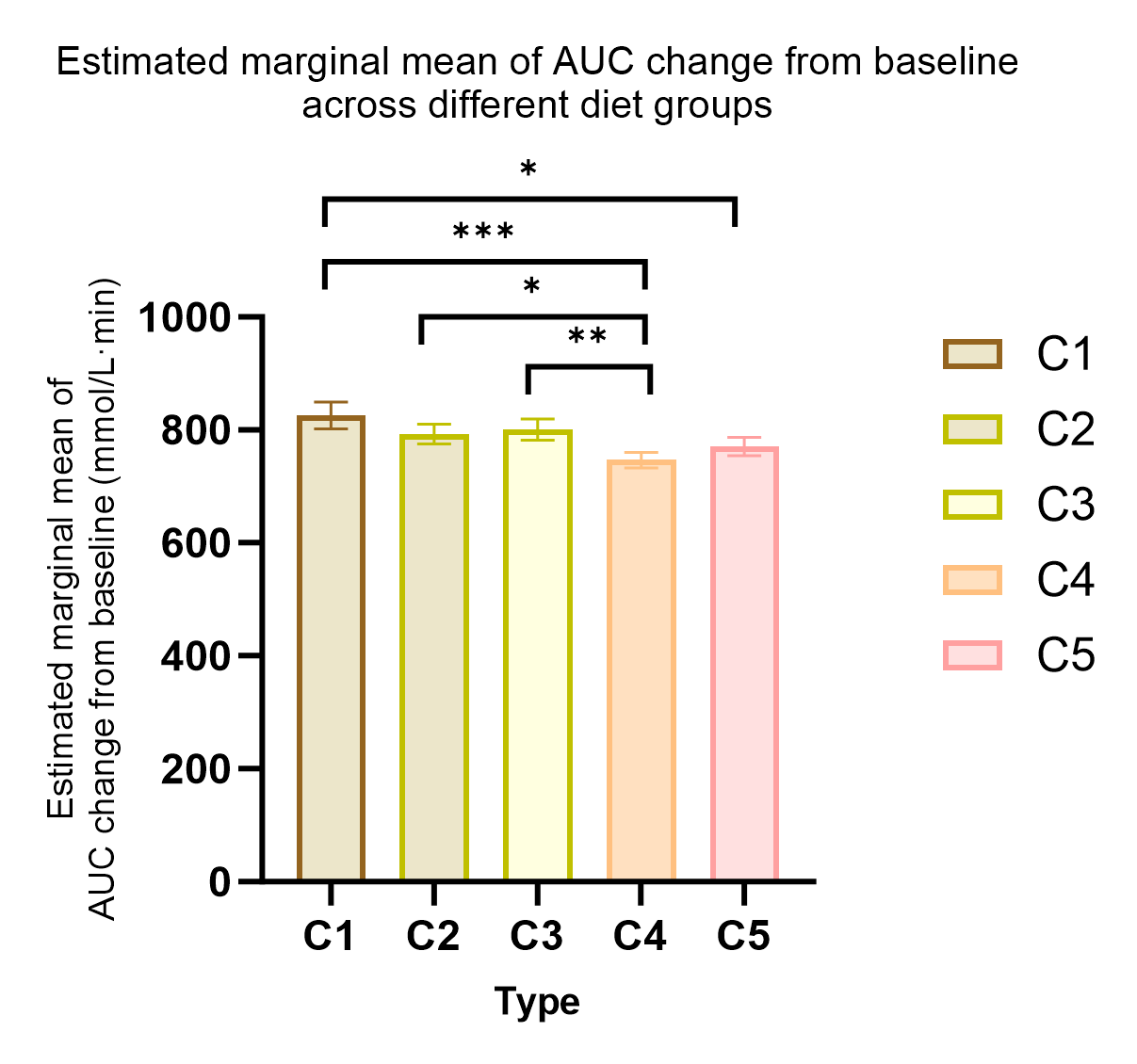


Figure 7 Comparison of estimated mean changes in postprandial 2 hour blood glucose AUC across different whole grain flour groups

Figure caption: C1—C5 represent buckwheat flour, millet flour, oat flour,yam flour, and soy milk powder, respectively

*: *P*< 0.05, **:*P* < 0.01, ***: *P* < 0.001

Figure 8 Comparison of estimated mean changes in postprandial 2 hour blood glucose iAUC across different whole grain flour groups

Figure caption: C1—C5 represent buckwheat flour, millet flour, oat flour,yam flour, and soy milk powder, respectively

*:*P* < 0.05,; **:*P* < 0.01, ***: *P* < 0.001


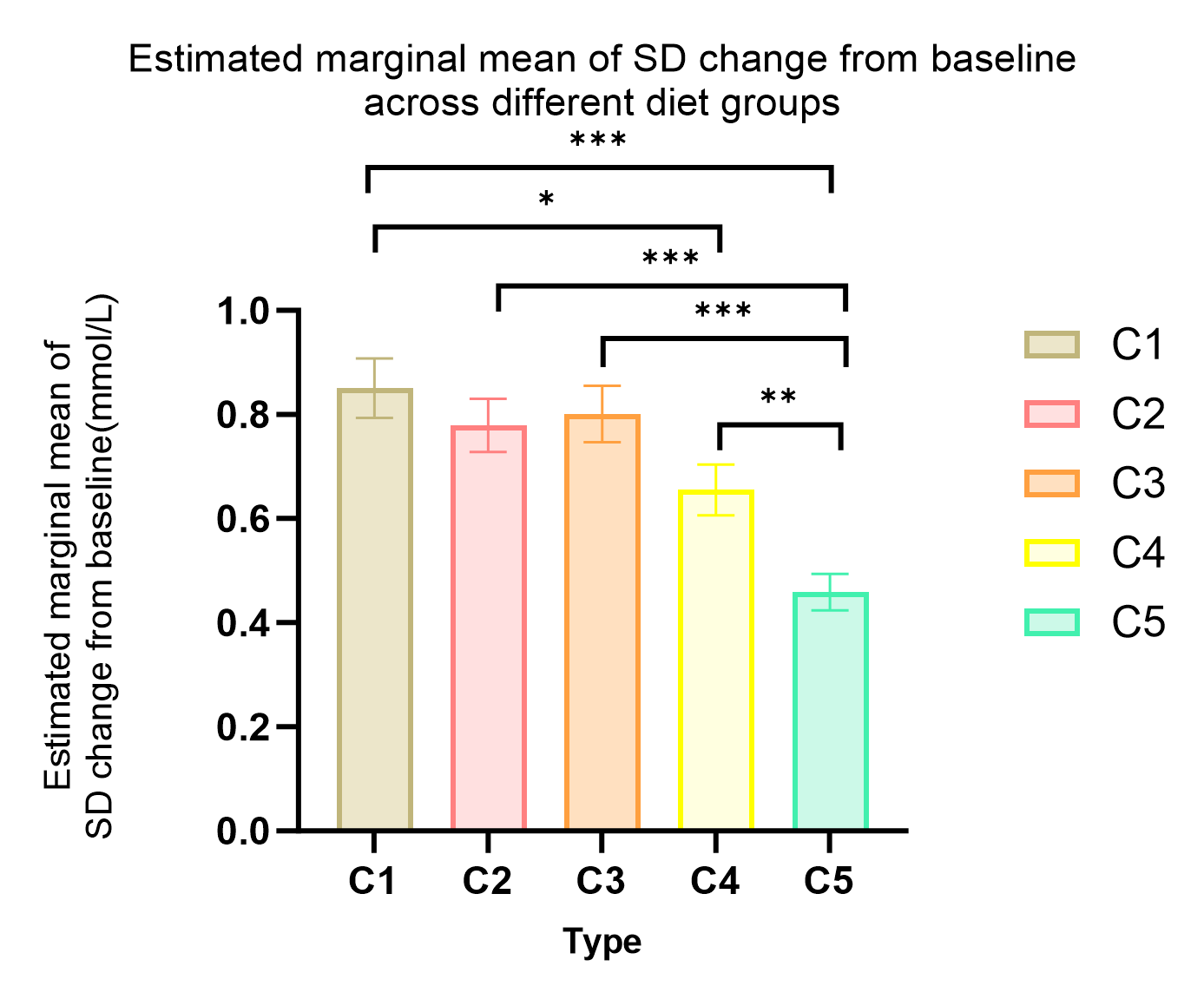


Figure 9 Comparison of estimated marginal means for postprandial 2 hour blood glucose SD across different whole grain flour groups

Figure caption: C1—C5 represent buckwheat flour, millet flour, oat flour,yam flour, and soy milk powder, respectively

*: *P* < 0.05, **:*P* < 0.01,***: *P*< 0.001

.
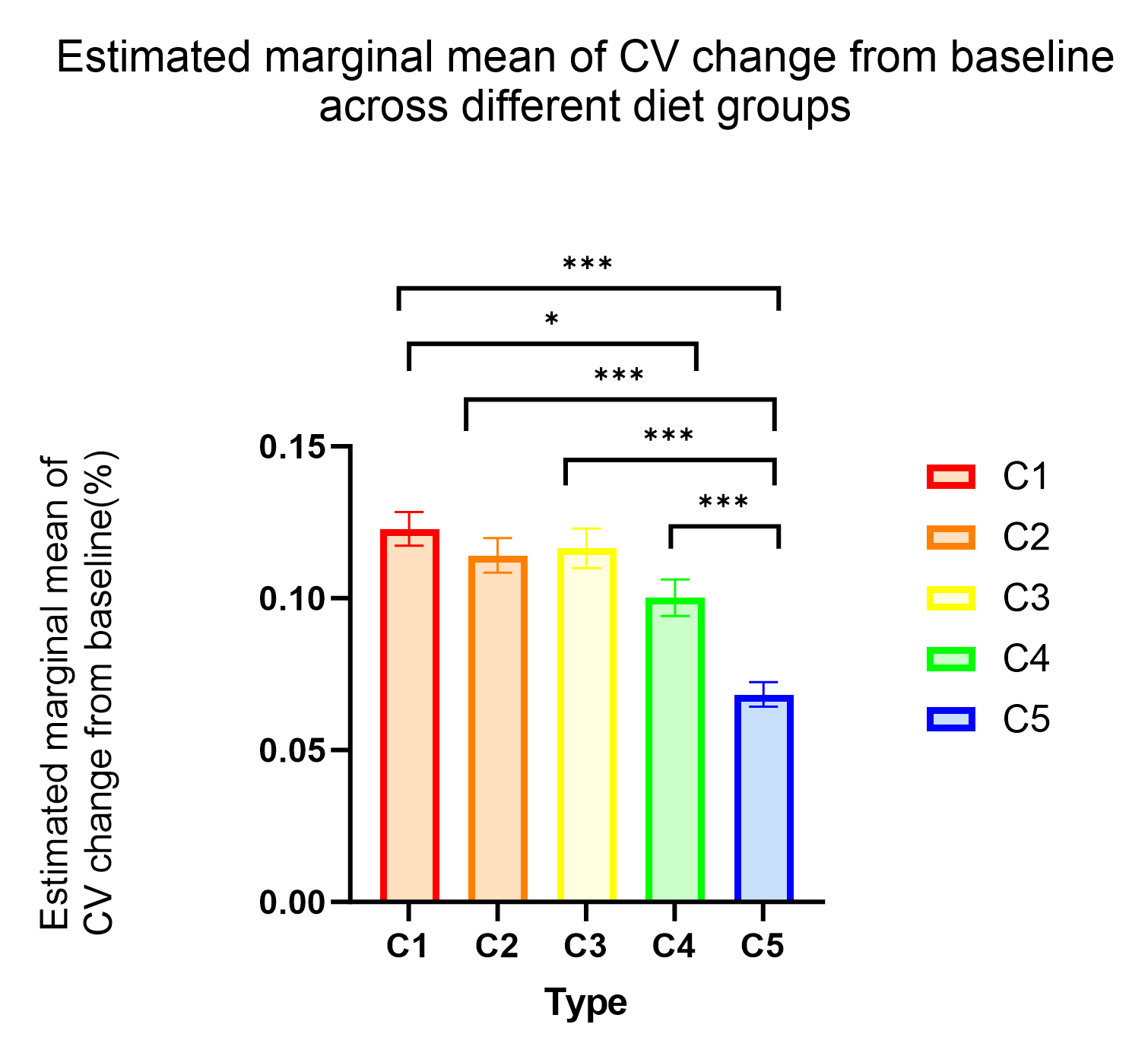


Figure 10 Comparison of estimated marginal means for postprandial 2 hour blood glucose CV across different whole grain flour groups

Figure caption: C1—C5 represent buckwheat flour, millet flour, oat flour,yam flour, and soy milk powder, respectively

*: *P* < 0.05,***:*P* < 0.001


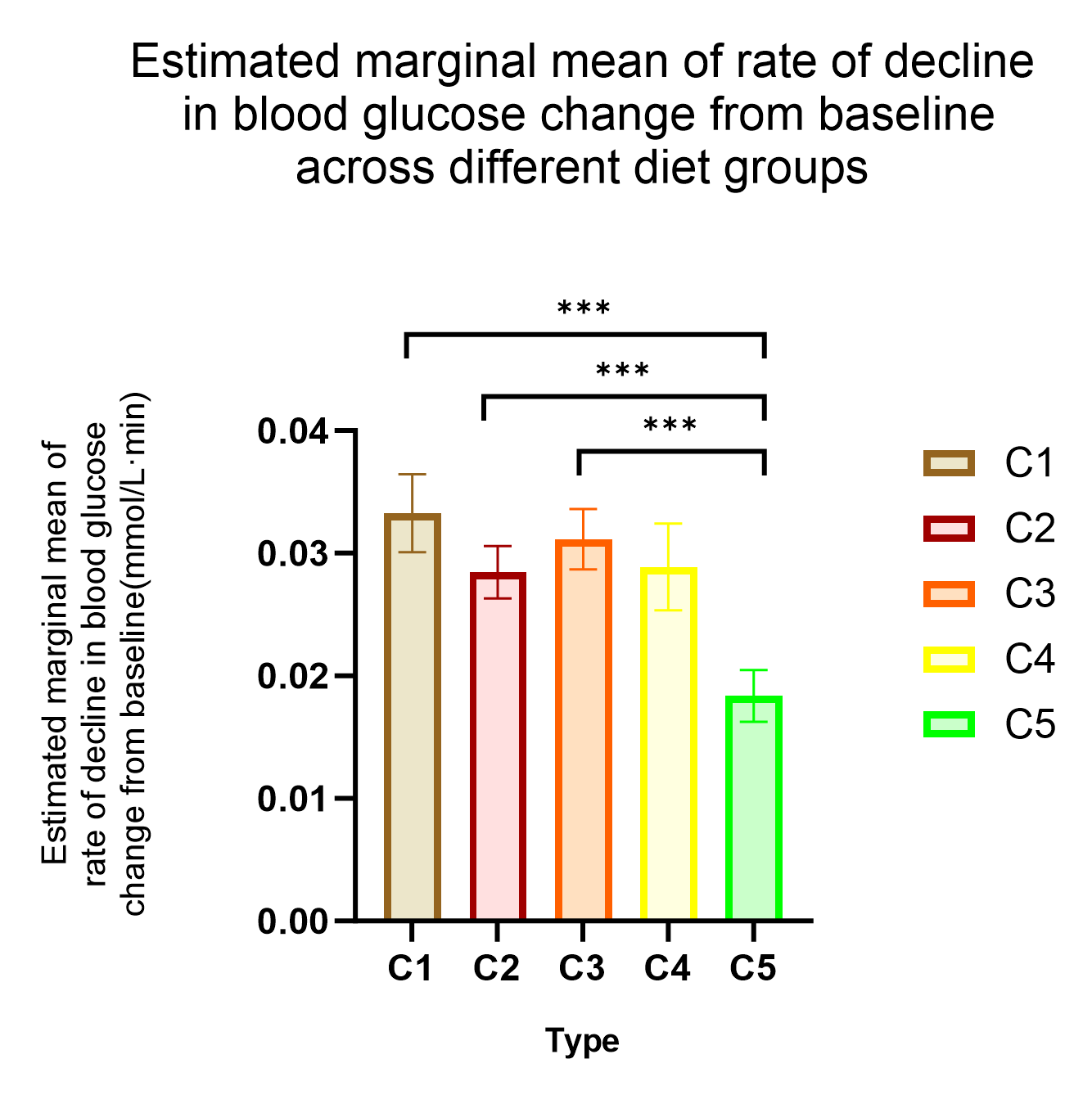


Figure 11 Comparison of estimated marginal means for the rate of postprandial blood glucose decline across different whole grain flour groups

Figure caption: C1—C5 represent buckwheat flour, millet flour, oat flour,yam flour, and soy milk powder, respectively

***: *P* < 0.001

Figure 12 Comparison of estimated marginal means of postprandial blood glucose rise rates 30 minutes after meals for different whole grain flours

Figure caption: C1—C5 represent buckwheat flour, millet flour, oat flour,yam flour, and soy milk powder, respectively

***:*P* < 0.001


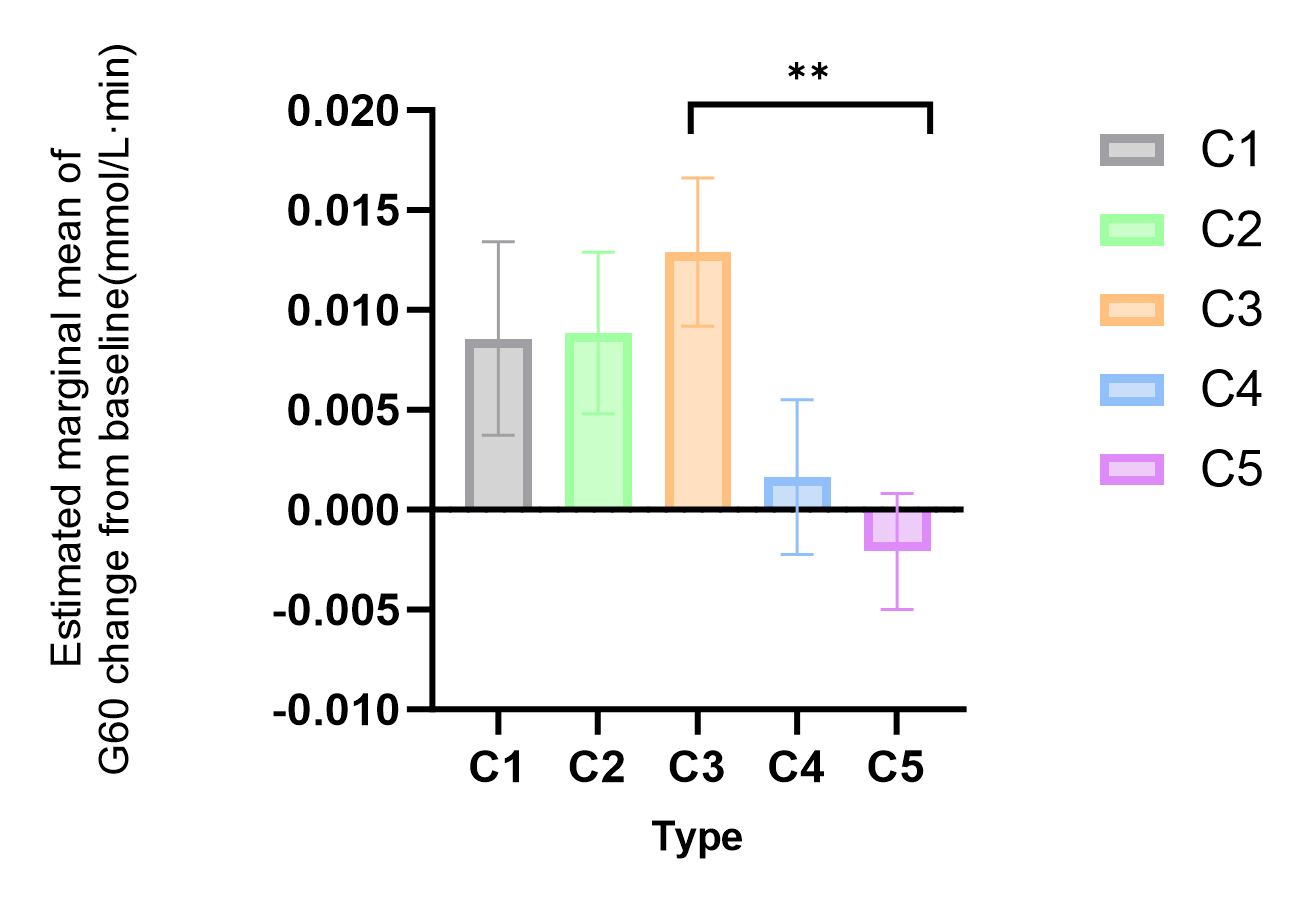


Figure 13 Comparison of estimated marginal means for the rate of postprandial blood glucose rise/fall in different whole grain flour groups

Figure caption: C1—C5 represent buckwheat flour, millet flour, oat flour,yam flour, and soy milk powder, respectively

**:*P* < 0.01

Figure 14 Comparison of estimated marginal means of the rate of decline two hours after a meal for different whole grain flour groups

Figure caption: C1—C5 represent buckwheat flour, millet flour, oat flour,yam flour, and soy milk powder, respectively

*:*P* < 0.05, ***:*P* < 0.001


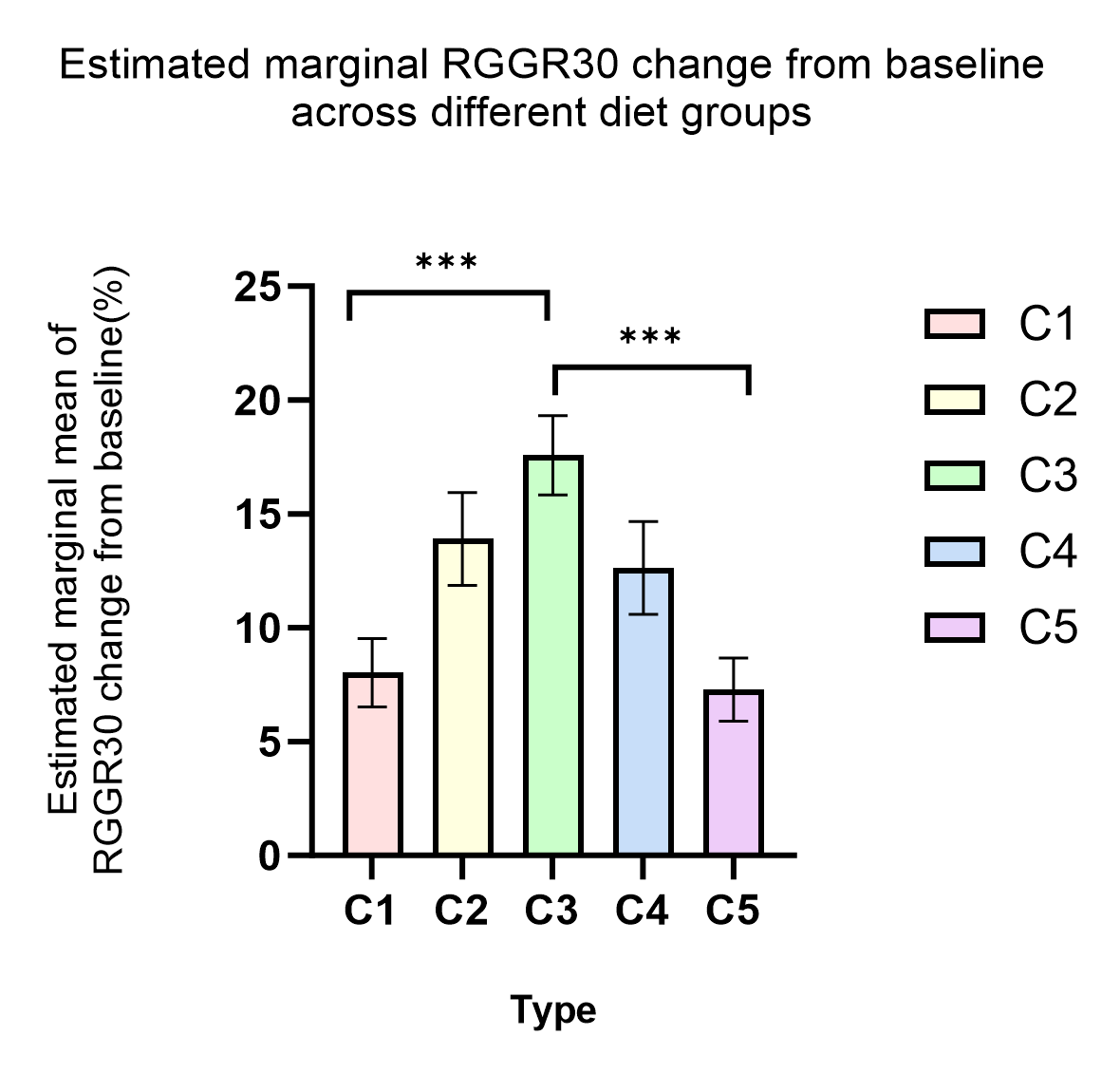


Figure 15 Comparison of estimated marginal means of the relative rise rate 30 minutes after a meal for different whole grain flour groups

Figure caption: C1—C5 represent buckwheat flour, millet flour, oat flour,yam flour, and soy milk powder, respectively

***: *P* < 0.001


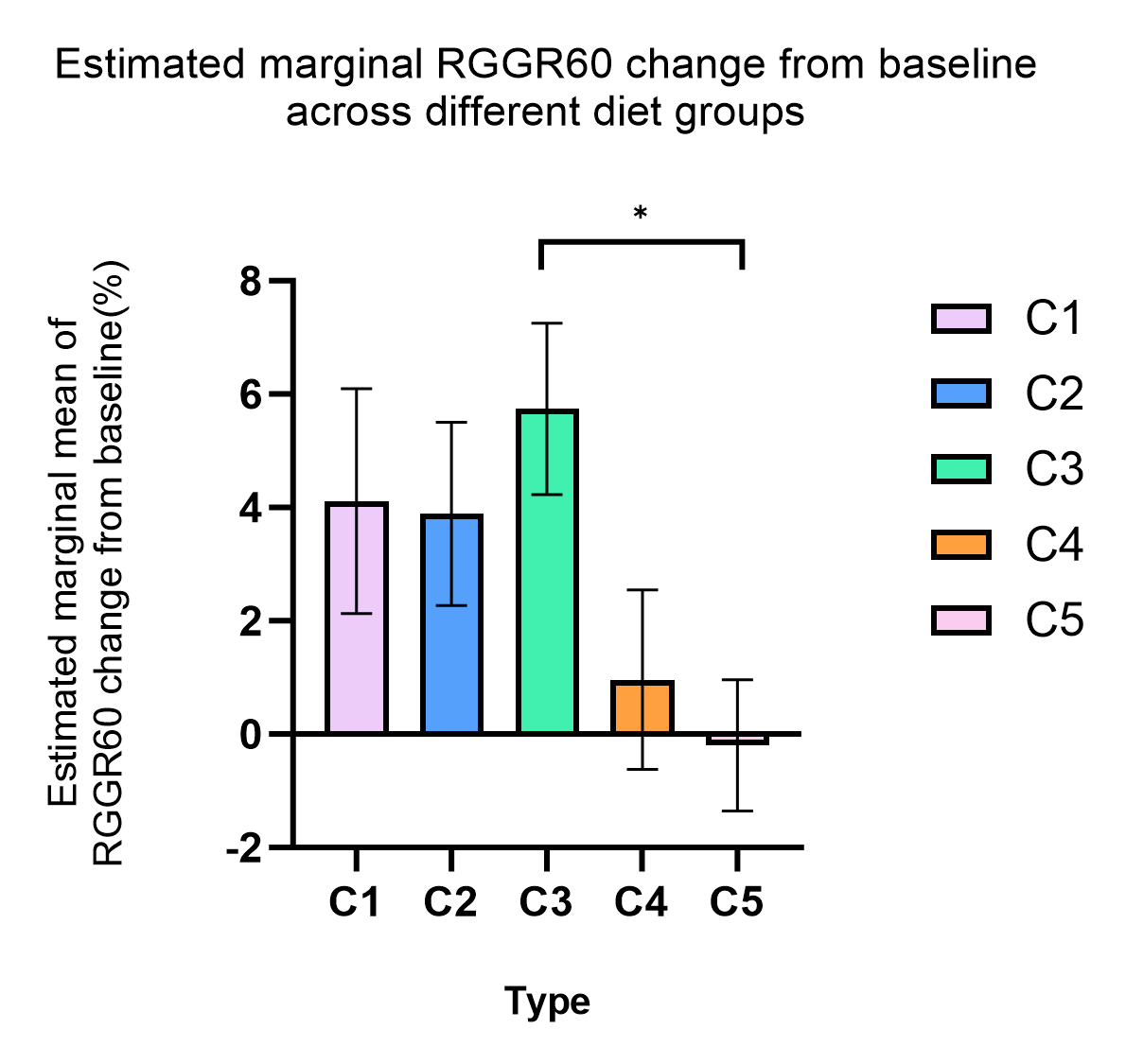


Figure 16 Comparison of estimated marginal means of relative rise/fall rates 60 minutes after meals for different whole grain flour groups

Figure caption: C1—C5 represent buckwheat flour, millet flour, oat flour,yam flour, and soy milk powder, respectively

*: *P* < 0.05; significant difference


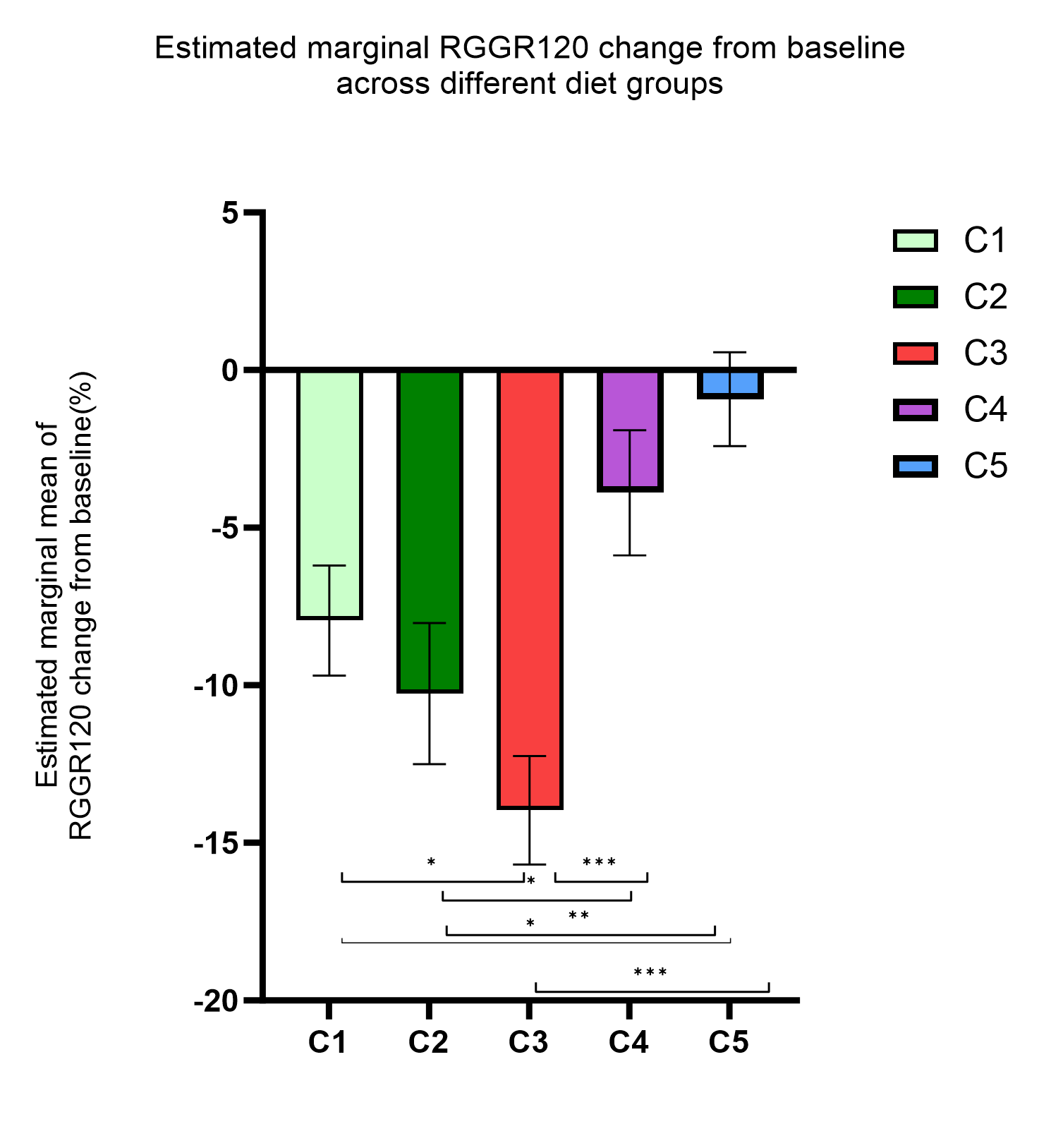


Figure 17 Comparison of estimated marginal means for the relative rate of decline 120 minutes after a meal across different whole grain flour groups

Figure caption: C1—C5 represent buckwheat flour, millet flour, oat flour,yam flour, and soy milk powder, respectively

*:*P* < 0.05, **: *P* < 0.01***: *P*< 0.001
